# Tunable Light-Responsive Polyurethane-urea Elastomer Driven by Photochemical and Photothermal Coupling Mechanism

**DOI:** 10.1021/acsami.4c00486

**Published:** 2024-04-06

**Authors:** Lei Wu, Xia Huang, Meng Wang, Jishizhan Chen, Jinke Chang, Han Zhang, Xuetong Zhang, Andrew Conn, Jonathan Rossiter, Martin Birchall, Wenhui Song

**Affiliations:** † Centre of Biomaterials for in Surgical Reconstruction and Regeneration, Department of Surgical Biotechnology, Division of Surgery & Interventional Science, University College London, London NW3 2PF, United Kingdom; ‡ School of Engineering and Materials Science, Queen Mary University of London, London E1 4NS, United Kingdom; § Suzhou Institute of Nano-tech and Nano-bionics, Chinese Academy of Sciences, Suzhou 215123, PR China; ∥ Dept of Engineering Mathematics and Bristol Robotics Laboratory, University of Bristol, Bristol BS8 1UB, United Kingdom; ⊥ UCL Ear Institute, Royal National Ear Nose and Throat and Eastman Dental Hospitals (UCLH NHS Foundation Trust), University College London, London WC1X 8EE, United Kingdom

**Keywords:** photoresponsive elastomer, photochemical stiffness softening, photothermal stiffness softening, nanophase separation, light-driven soft robotics, untethered bionic fingers, polyurethane elastomer actuator

## Abstract

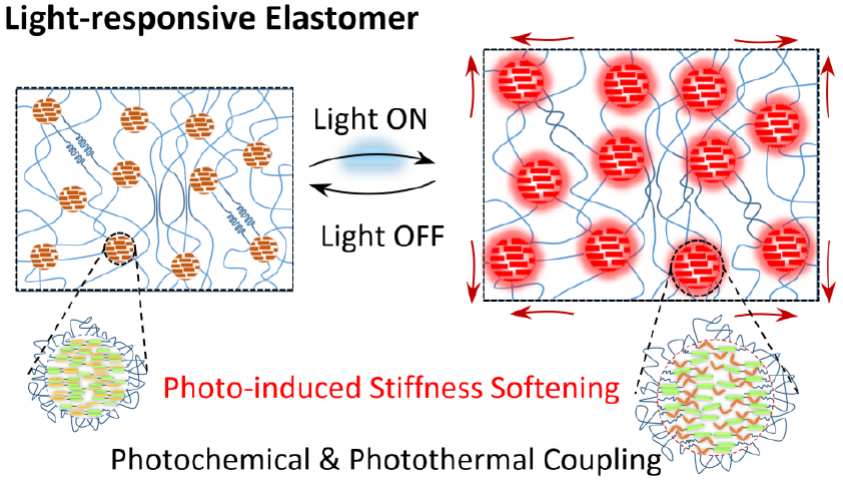

Light-driven soft actuators based on photoresponsive materials can be used to mimic biological motion, such as hand movements, without involving rigid or bulky electromechanical actuations. However, to our knowledge, no robust photoresponsive material with desireable mechanical and biological properties and relatively simple manufacture exists for robotics and biomedical applications. Herein, we report a new visible-light-responsive thermoplastic elastomer synthesized by introducing photoswitchable moieties (i.e., azobenzene derivatives) into the main chain of poly(ε-caprolactone) based polyurethane urea (PAzo). A PAzo elastomer exhibits controllable light-driven stiffness softening due to its unique nanophase structure in response to light, while possessing excellent hyperelasticity (stretchability of 575.2%, elastic modulus of 17.6 MPa, and strength of 44.0 MPa). A bilayer actuator consisting of PAzo and polyimide films is developed, demonstrating tunable bending modes by varying incident light intensities. Actuation mechanism via photothermal and photochemical coupling effects of a soft–hard nanophase is demonstrated through both experimental and theoretical analyses. We demonstrate an exemplar application of visible-light-controlled soft “fingers” playing a piano on a smartphone. The robustness of the PAzo elastomer and its scalability, in addition to its excellent biocompatibility, opens the door to the development of reproducible light-driven wearable/implantable actuators and lightweight soft robots for clinical applications.

## 1 Introduction

Conventional rigid robots can replace human work by carrying out a series of complex actions. However, a lack of multiple-sensing and softness in materials and adaptability in motions limits their application in unpredictable or harsh environments including direct biological contact and human–machine interfaces. Inspired by nature, an emergent range of soft robots, made from mechanically compliant and stimulus–responsive polymers, have been shown to mimic biological motor systems. Unlike rigid robots, soft robots are more adaptive and flexible with respect to biological and potential medical environments and lack bulky actuators. Thus, prototypes of soft robotic hands and sensitive artificial skin have been developed,^1−3^ as well as responsive implants for controlled targeted cell/drug delivery and minimally invasive surgery soft robotic tools.^4,5^

Light-driven soft robotic actuators based on photoresponsive materials have replicated swimming,^6−8^ walking,^9−14^ crawling,^15−19^ rolling,^10,20−22^ gripping,^23,24^ jumping,^25−27^ and oscillating.^19,22,28−31^ Light-driven soft robots have uniquely attractive advantages for potential healthcare, food, and agriculture applications: they offer rapid, dexterous and precise spatial and temporal remote control,^32−34^ and ease of miniaturization, and they are low in weight.^32,35−38^ Furthermore, light-responsive materials have a broad range of tailorable properties which can be matched to specific biological settings.^37,39,40^ However, photoresponsive materials reported to date require time-consuming and expensive development including molecular designs, complex monomers, and polymer synthesis and manufacturing techniques. Majority of photoresponsive polymers reported are cross-linked thermoset liquid crystalline elastomers requiring prealignment for maximizing anisotropic performance and a nonreprocessable and nonrecyclable cross-linking process (Table S1). Clearly, new light-responsive materials that can render highly robust and controllable photomechanical properties and simple processability would be of great value as the basis of potential solutions to unmet biomedical needs.

Azobenzene derivatives (“azobenzenes”) are the most widely used photoresponsive molecules for converting light into mechanical deformation,^41^ during which the *trans*–*cis* isomerization of two isomeric states, a rod-like *trans* state and a bent-shape *cis* state, occurs by UV light irradiation. Azobenzenes are widely used as building blocks for synthesis of photoresponsive polymers, covalently bonded into different types of linear, 3D network, or dendrimer chain architectures.^32,33,42,43^ Cross-linked liquid crystalline polymers (CLCPs) consist of azobenzene-mesogens in both main chains and side chains of polyacrylate, capable of directional bending and generation of continuous, directional, macroscopic mechanical waves under constant light illumination.^36,44^ A new family of azobenzene-containing rotaxane-branched dendrimers capable of controlling reversible movements from nano- to macroscale has been reported. However, the robustness, stability, and cytotoxicity of these polymers have not been fully investigated. Their intrinsic environmental stability, water resistance, and hyperelasticity (mechanical strength and stretchability) are often inferior to those of many other polymers, and their irreversible cross-linking process of CLPCs, low mechanical properties of the dendrimers, and high production costs may limit their biomedical applications, especially for assistive and implantable devices. Among synthetic polymers, thermoplastic polyurethane (PU) elastomers have been widely used for medical applications due to their versatile macromolecular design and synthesis, as well as tunable hyperelasticity, outstanding fatigue resistance, and biocompatibility.^45,46^ The introduction of azobenzenes and other photoresponsive molecules into PU chains may offer a new group of photoresponsive elastomers with desired mechanical properties and biocompatibility, as indicated in some early and recent studies,^43,47^ which can potentially fulfill the challenging demands for biomedical applications.

In parallel, the study of stimuli-induced mechanical behaviors such as stiffness softening is necessary.^48^ We demonstrated that a thermal-induced stiffness softening of 3D-TIPS polyurethane scaffolds at body temperature was due to the melting phase transition of a small fraction of crystals of the soft segments.^48−50^ Unlike this thermally induced stiffness softening by temperature, light-driven stiffness softening involves more complex underpinning mechanisms: photochemical and photothermal effects. Photochemically, the photoisomerization of azobenzene alters the glass transition temperature (*T*_g_) of azopolymers, in turn generating the mobility of the polymer chains and resulting in softening. Notably, photoswitched glass transitions were reported in azopolymers with azobenzene groups grafted as side-chains^51^ and cross-linked within a liquid crystalline polymer network.^52^ The *trans*-to-*cis* isomerization of azobenzene mesogens was proven to trigger the glass transition of chain segment relaxation within liquid crystalline networks, enabling photomechanical responses at room temperature under UV/visible light.^52^ However, the coupling photothermal effect generated by light has been less studied. In fact, a proportion of light absorbed by polymers is converted into heat, leading to softening at higher temperatures.^44,53^ Despite increasing efforts to develop more light-responsive elastomers and their applications, the underlying photomechanism is still not fully understood in both theoretical and experimental domains. An in-depth understanding and quantification of the combined influence of both photochemical and photothermal effects on the stiffness softening behaviors of photoresponsive elastomers have become pivotal if their promise for a new generation of soft actuators and robotics for biomedical applications is to be realized.

**Figure 1:**
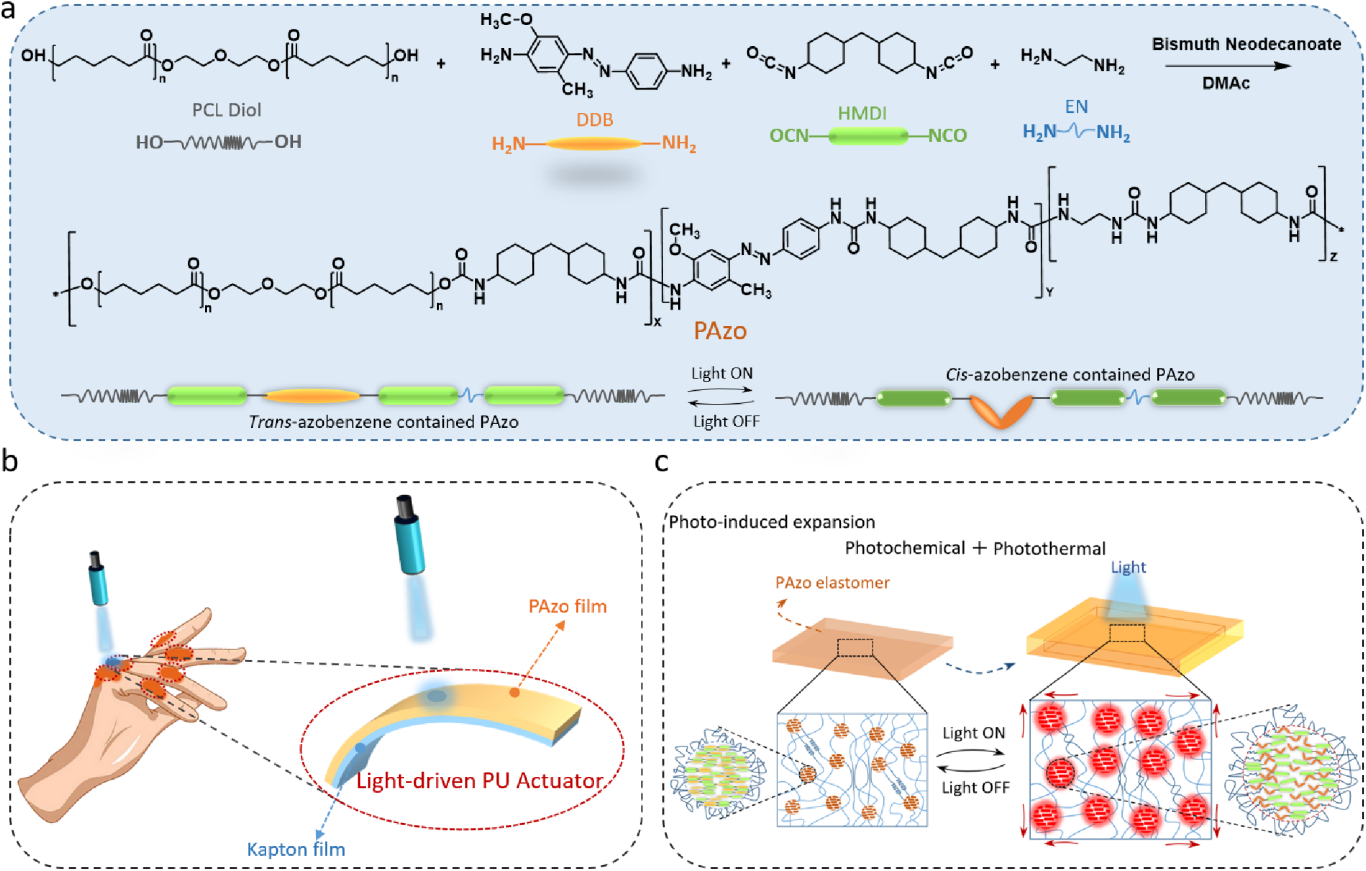
(a) Synthesis pathway for azobenzene-containing poly(urethane urea) elastomer (PAzo); (b) schematic diagram of a light-driven PAzo actuator for putative hand-assisted application; (c) schematic diagram of the photochemical/photothermal effect (photoinduced expansion) for the PAzo elastomer.

Here, we describe a novel visible-light-responsive thermoplastic elastomer (PAzo) synthesized by introducing azobenzene derivative photoresponsive molecules into the backbone of poly(ε-caprolactone) and aliphatic 4,4-methylenebis(cyclohexyl isocyanate) based polyurethane urea (PCL–PUU) (Figure 1a). The synthesized PAzo polymer responded to blue light and exhibited excellent hyperelasticity (elastic modulus of 17.6 MPa; ultimate strain of 575.2%; strength of 44.0 MPa) and good biocompatibility. A visible-light-driven bilayer actuator consisting of PAzo and Kapton polyimide sheets (Figure 1b) was designed and fabricated, and its actuation performance and mechanisms were explored. Specifically, two bending modes of the actuator were observed and characterized under illumination with varying light intensities. Photochemical and photothermal coupling actuation mechanisms (Figure 1c) were studied experimentally and theoretically. Finally, we demonstrated the bilayer actuator in the form of soft robotic fingers for playing a touch piano on a smartphone.

## 2 Results and Discussion

### 2.1 Synthesis, Mechanical, and Biological Performance of PAzo

As shown in Figure 1a, polycaprolactone (PCL) diol, 4,4′-methylenebis(cyclohexyl isocyanate) (HMDI), disperse diazo black 3BF (DDB, photoresponsive molecule), and ethylenediamine (EN, a chain extender) were used to synthesize PAzo (two-step polymerization, see Section 4) and polymer films cast for further characterization. As a comparator, PCL–PUU (without azobenzene) was also synthesized (details in the Supporting Information, adapted from previous work).^54^ It is of note that, in this study, the reaction ratios of PCL diol, DDB, and EN with HMDI were optimized based on their impact on the structure and properties of the resulting PAzo elastomers, which will be featured in a future paper.

**Figure 2:**
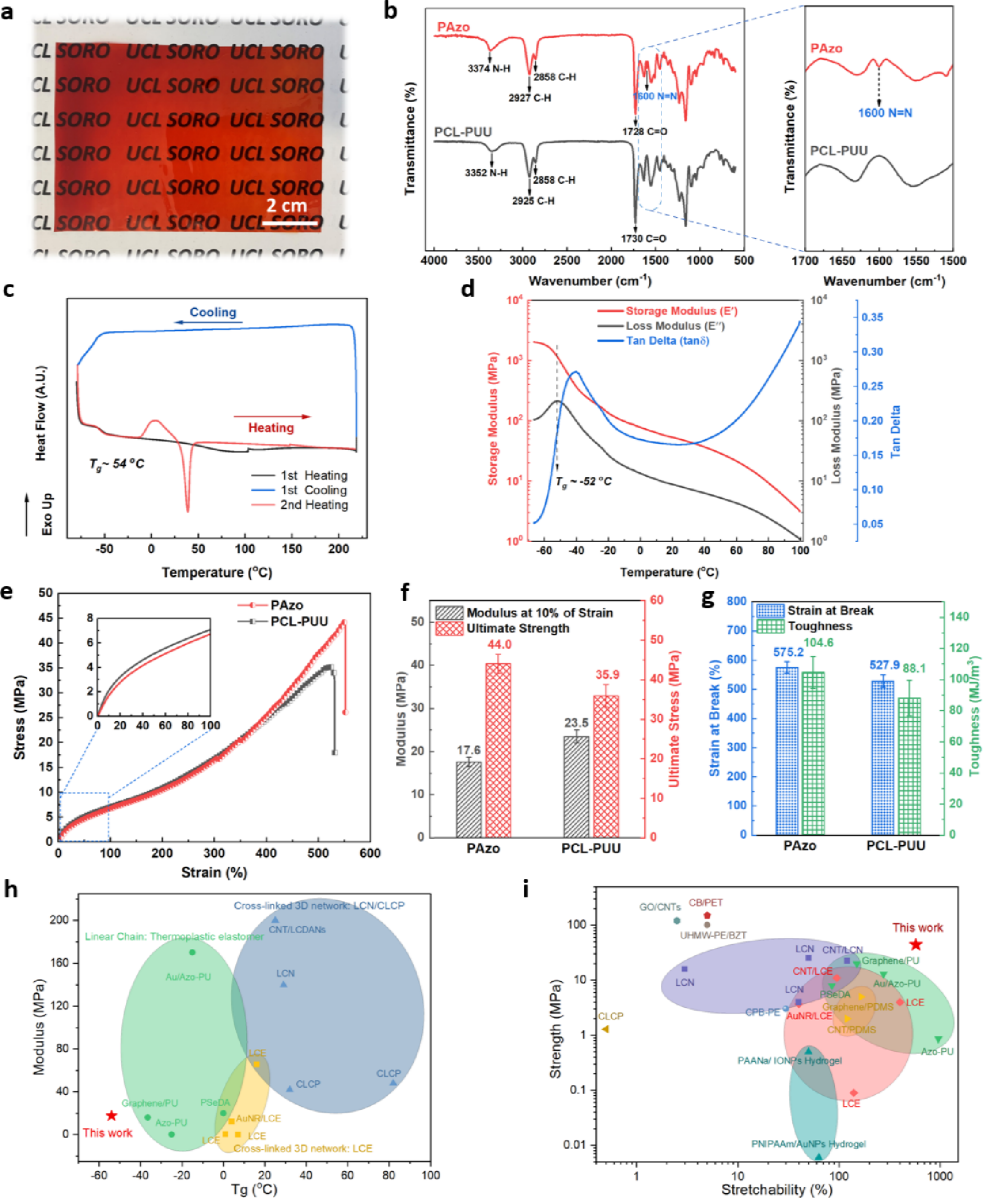
Physiochemical characterizations of PAzo. (a) Photo of a transparent PAzo cast sheet; (b) FTIR spectra of PAzo and PCL–PUU films (enlarged spectra displaying the absorption peak at 1600 and 1509 cm^–1^ corresponding to the C=C and *N*=N vibration of azobenzenes respectively); (c) DSC temperature profiles of PAzo film in the range from −80 to 220 °C (first heating and cooling and second heating ramps); (d) dynamic mechanical properties as a function of temperature at a frequency of 1 Hz (glass transition temperature of around −52 °C); (e) stress–strain curves of PAzo and PCL–PUU (insert diagram shows the stress–strain curve of the first 0–100% strain); (f) tensile modulus (at 10% of strain) and ultimate strength, (g) strain at break and toughness of PAzo and PCL–PUU; Comparisons between PAzo elastomer and other existing photoresponsive materials: (h) Ashby plot of modulus versus glass transition temperature (*T*_g_) of photoresponsive materials including a thermoplastic elastomer^47,55−57^ with linear chain architectures and cross-linked LCE^16,52,58,59^/LCN^52,60^/LCP;^61^ (i) Ashby plot of strength versus stretchability of the PAzo elastomer and other photoresponsive materials including thermoplastic elastomers,^47,55−57^ hydrogels,^27,31^ LCE,^16,20,58^ PDMS,^22,62^ LCN,^30,52,60,63^ CLCP,^61^ GO/CNTs,^21^ CB/PET,^9^ UHMW-PE/BZT,^64^ CPB-PE,^11^ data from Table S1.

The PAzo cast film exhibits a transparent orange-red color (Figure 2a), while PCL–PUU is clear transparent (Figure S1). The FTIR spectra of PAzo and PCL–PUU are largely similar (Figure 2b) and do not show the stretching vibration band of isocyanate (—N=C=O) groups at around 2273 cm^–1^, indicating the completion of the reaction with the —N=C=O groups of HMDI. Both spectra also display absorption peaks at wavenumbers around 3370, 2927/2858, and 1730 cm^–1^, attributed to N–H, C–H, and C=O vibrations, respectively. However, the spectrum of PAzo shows obvious peaks at around 1600 and 1509 cm^–1^ (enlarged picture in Figure 2b), which corresponded to the C=C and *N*=N vibrations of azobenzenes bonded to the main chains of PAzo, respectively. These results indicate that PAzo and PCL–PUU were successfully synthesized. The glass transition temperature (*T*_g_), at around −54 °C, of the PAzo cast film is clearly observed from the first heating ramp by differential scanning calorimetry (DSC, Figure 2c), and no other thermal transition peaks up to 220 °C were observed, suggesting a rubber phase in a broad range of temperature before thermal degradation occurred at around 250 °C as measured by TGA (Figure S2). Interestingly, an exothermic crystallization peak at 5 °C and an endothermic melting peak with *T*_m_ at around 38 °C appeared during the secondary heating ramp, suggesting that recrystallization and melting of PCL soft segments may be involved under suitable thermal conditions, in the vicinity of human body temperature (details of the analysis are listed in Table S2 and Figure S3).

The temperature ramp tested by a dynamic mechanical analyzer (DMA) in Figure 2d demonstrates marked changes in the viscoelastic behavior of PAzo at around the *T*_g_ (∼−52 °C) and *T*_m_ (∼38 °C) of PCL soft segments of PAzo, consistent with those observed in the DSC ramp (Figures 2c and S3). The storage modulus, *E*′, of PAzo dropped dramatically while the loss modulus, *E*″, peaks during the glass transition. The falls of both *E*′ and *E*″ slowed in the rubber phase after the increase of *T*_g_ and then dramatically decreased again with a sharp increase in tan δ when the temperature reached the *T*_m_ of PCL soft segments. This suggests a pronounced increase in chain relaxation and viscous properties during the phase transition from the semicrystalline phase to the amorphous rubber phase of the soft domains.

To evaluate the static mechanical performance of the PAzo film, a uniaxial tensile test was performed. As shown in Figure 2e–g, PAzo shows a typical hyperelastic stress–strain relationship, similar to that seen with PCL–PUU. As expected, the Young’s modulus within the first linear elastic region of 10% strain was measured at around 17.6 MPa, which is slightly lower than that of PCL–PUU (23.5 MPa). By contrast, PAzo’s ultimate strength (44.0 MPa), fracture strain (575.2%), and toughness (104.6 MJ/m^3^) are superior, compared to 35.9 MPa, 527.9%, and 88.1 MJ/m^3^ for PCL–PUU. Regardless, the stress–strain behaviors suggest that PAzo inherits the characteristics of a poly(urethane urea) elastomer (PCL–PUU): highly flexibility and stretchability because of unique self-assembling interactions and nanophase separation of its covalently bonded soft and hard segments resulting marked entropic elasticity. Here, we note the existence of azobenzene linkages (rigid —N=N— double bonds connecting two phenyl rings) as part of hard segments that contribute importantly to the strength, modulus, and toughness of PAzo elastomer films.

Photoresponsive materials may be classified into two categories based on their polymer chain architectures: linear chain thermoplastic elastomers and 3D cross-linked liquid crystal polymeric elastomers (LCE/LCN/LCP). The dramatic changes in moduli of these materials are thought to depend on their glass transition temperature (*T*_g_), which is determined by the mobility of the polymer chain segments.^51,52^Figure 2h shows an Ashby plot of the modulus and *T*_g_ of various photoresponsive materials reported in the literature. The polyurethane urea elastomer, PAzo, synthesized in this work, with its long linear chains, has the lowest *T*_g_ at approximately −54 °C among most photoresponsive LCN/LCP materials. As above, the polymer is in the rubber phase over a wide range of temperatures, from approximately −54 to 220 °C (Figure 2d). The modulus of the PAzo elastomer, 17.6 MPa at room temperature, is one of the lowest among those of most LCN/LCP materials (>100 MPa), making it more suitable for soft wearable robotic applications with ultrahigh compliance over a wide range of temperatures, for application even below freezing conditions.

**Figure 3:**
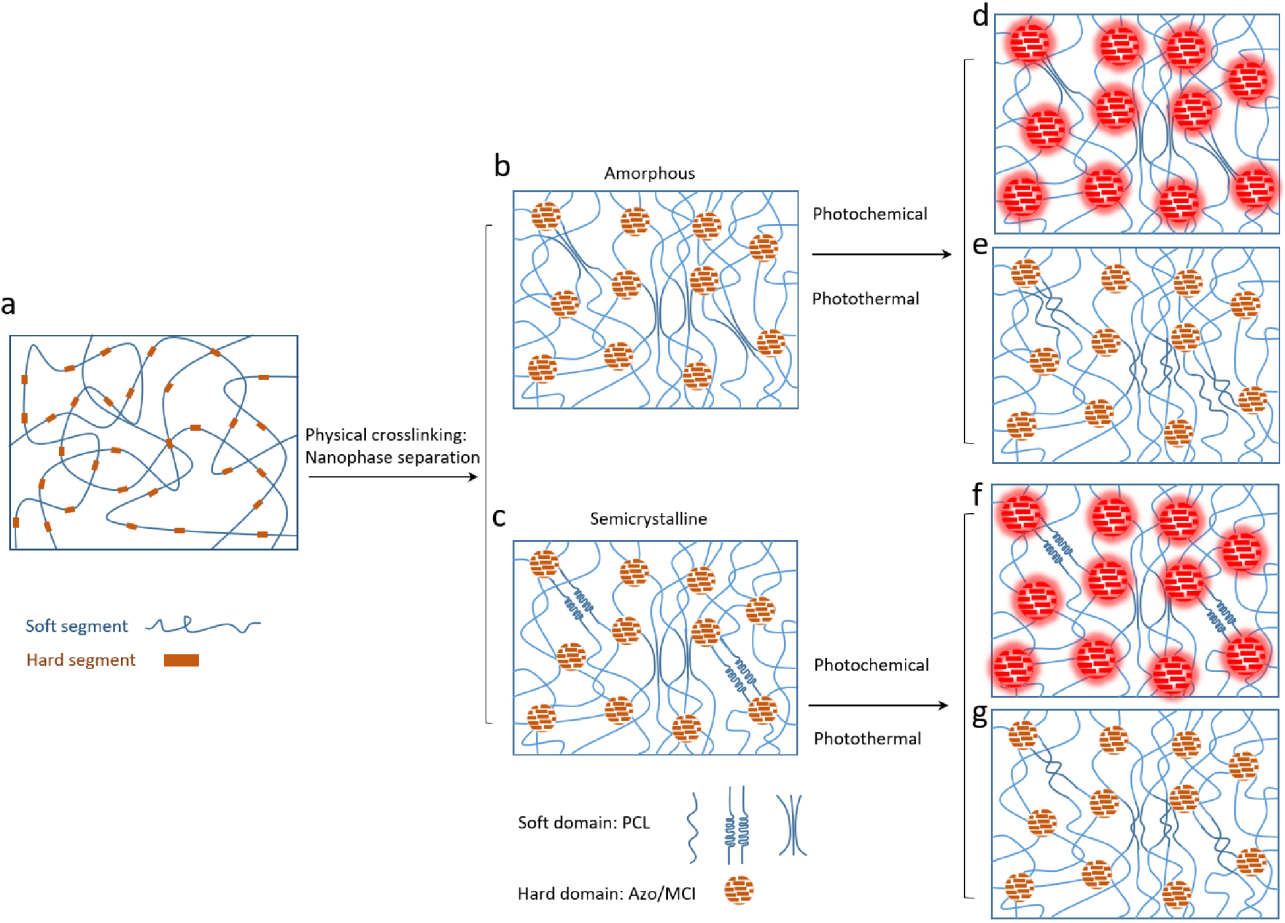
Self-assembly and nanophase structure of PAzo elastomer, and its photoresponsive mechanisms: (a) random coiled chain conformation of PAzo elastomer in solution; (b and c) nanophase structure of PAzo elastomer solid with amorphous (b) and semicrystalline (c) phase of the soft domain; (d–g) The nanophase structure change of amorphous and semicrystalline PAzo elastomers induced by photochemical (d and f) and photothermal (e and g) effects, respectively.

Polymer materials can fail when they reach either their ultimate strain or their ultimate strength. The high stretchability and strength of a photoresponsive polymer are critical for use in actuators. Photoresponsive materials present a unique challenge in this regard, as there is often a trade-off between their stretchability and strength as shown in the Ashby plot in Figure 2i. For instance, while LCEs are stretchable, they cannot withstand high stress due to their low degree of cross-linking. As a result, LCNs are relatively strong and stiff but less extensible due to their 3D cross-linked networks with limited chain mobility in either the rubber or glassy state. In this study, the photoresponsive linear-chain PAzo elastomer exhibits both high strength (44 MPa) and notable stretchability (575.2%), compared with other photoresponsive materials as shown in Figure 2i. The exceptional mechanical performance of the PAzo elastomer may be attributed to its well-known unique nanophase separations of hard–soft domains through self-assembling (Figure 3a–c). A more detailed comparison of the PAzo elastomer and other photoresponsive materials, including the material type, *T*_g_, *T*_m_, T_i_, photomechanics principles, chain architectures, modulus, stretchability, tensile strength, and actuation stress is presented in Table S1. The excellent biocompatibility of PAzo in promoting cell adhesion and proliferation was also confirmed by *in vitro* studies (Figure S4).

### 2.2 Photochemical and Photothermal Mechanics of PAzo

**Figure 4:**
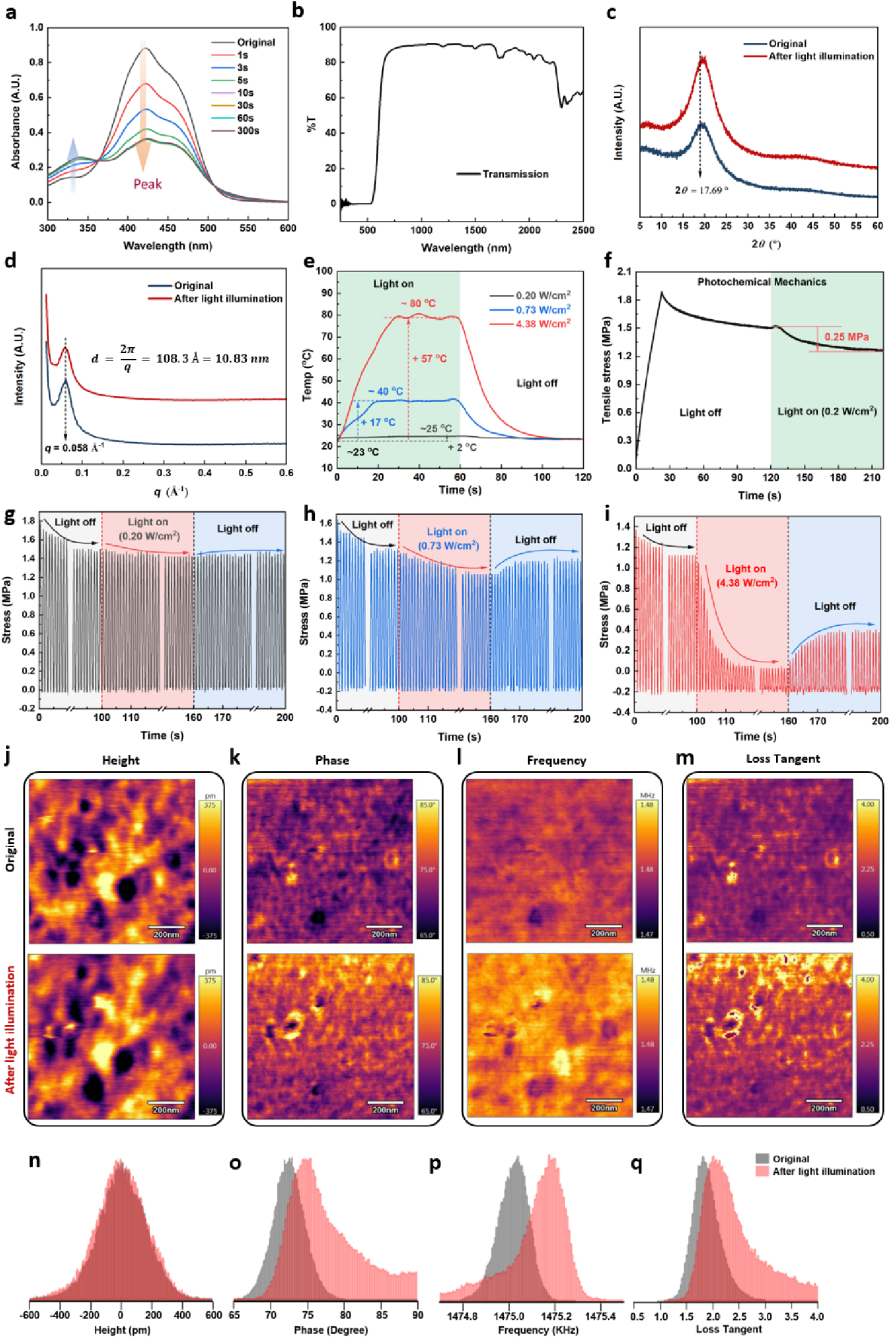
(a) UV–vis absorption spectra of PAzo solution when irradiated by 470 nm light (0.20 W/cm^2^) for varying time periods; (b) transmission spectrum of PAzo film determined by a Universal Measurement Spectrophotometer (UMS) across the spectral range from 250 to 2500 nm; (c) WAXD and (d) SAXS curves of PAzo film before and after light-illumination (470 nm light with an intensity of 4.38 W/cm^2^ for 60 s); (e) temperature changes of PAzo film when irradiated by 470 nm light with different intensities; (f) tensile stress changes of PAzo film (with stretching and annealing treatment) when irradiated with 470 nm light (0.2 W/cm^2^) (sample dimension of 20 × 5 × 0.15 mm^3^); dynamic mechanical stress changes of PAzo when irradiated by light of different intensities (g) 0.20 W/cm^2^, (h) 0.73 W/cm^2^, and (i) 4.38 W/cm^2^ (dynamic test conditions: room temperature, 10% strain, 1 Hz, light off 100 s, on 60 s then off 40 s, sample dimension of 10 × 2 × 0.18 mm^3^); atomic force microscopy (AFM) (j) height, (k) phase, (l) frequency, and (m) loss tangent images of original PAzo (top) and PAzo after *in situ* light illumination (bottom) (470 nm light, 0.2 W/cm^2^), and corresponding histogram distribution changes of (n) height, (o) phase, (p) frequency, and (q) loss tangent.

It is well acknowledged that azobenzenes undergo reversible photochemical reactions; in that, *trans*-to-cis isomerization occurs upon irradiation with UV light and can reverse to their initial configuration via cis-to-*trans* isomerization by thermal relaxation or visible-light irradiation.^33^ Here, PAzo can be triggered by both UV and 470 nm blue light and reversed from cis-to-*trans* configuration when relaxed under dark conditions for around 2h (Figures 4a and S5a). As shown in Figures 4a and S5c, after irradiation with UV or blue light (365 or 470 nm, light intensity of 0.20 W/cm^2^), the absorption peaks between 400 and 470 nm, characteristic of *trans* azobenzene, show a significant decrease from *t* = 1 to 300 s, while the absorbance in the UV light region (∼330 nm, indicative of *cis* azobenzene) increases. By contrast, PCL–PUU (without covalent bonding with azobenzene) did not show any clear absorption when irradiated by 365 or 470 nm LED light (Figure S5d). As shown in Figure 4b, the PAzo film exhibits relatively high transmission across the near-infrared (NIR) region before a sharp decrease around 800 nm, which falls to around 0% of the transmission at about 530 nm. This demonstrates that the PAzo film has high transparency in a broad band from visual to infrared light.

As with other thermoplastic poly(urethane urea) elastomers, both hyperelasticity and viscoelasticity of PAzo are related to its self-assembled nanophase structure, indicated by wide-angle X-ray diffraction (WAXD), small-angle X-ray scattering (SAXS), and dynamic mechanical mapping at the nanoscale by AFM. It is well-known that the soft segments and hard segments of long linear macromolecular chains self-assemble into soft domains and hard domains at the nanoscale, where soft domains, often in the rubber phase, provide superior entropic elasticity while hard domains in the glassy solid phase serve as physical cross-linking points to reinforce the strength and stiffness (Figure 3b).^50^ On the other hand, its phase structure and physical properties, such as crystallinity or phase transition temperature, can be tailored by the composition of its soft and hard segments as well as by the fabrication processes itself (thermal treatment).^65^ Notably, in the case of PAzo, the soft segments of PCL tend to crystallize easily at around 5 °C and melt again at around 38 °C during cooling or heating with and without light irradiation (∼1 W/cm^2^ for 1 min), as determined by DSC (Figures 2b and S3). In Figure 4c, WAXD analysis shows a broad, diffuse peak at 2θ = 19.95°, suggesting a predominantly soft, amorphous rubber-phase structure of the PAzo film (Figure 3b). The peak sharpens after light illumination, indicating more physical entanglements and/or a slight recrystallization of PCL soft segments during cooling (Figure 3c). This is represented by a shoulder peak at 2θ = 17.69° (*d*_spacing_ = 0.50 nm according to the Bragg equation), corresponding to the crystalline structure of the PCL soft domain^66^ (detailed *d*_spacing_ results in Figure S6 and Table S3). This conclusion is also supported by a relatively large decrease in the strain of PAzo during cooling, related to partial crystallization of PCL soft segments (Figure S7). The SAXS spectra of PAzo films (Figures 4d and S6) show, despite soft segment nanophase transition, that the broad peaks corresponding to the long spacing of 10.83 nm between the hard segment nanodomains remained unaltered by light stimulation.

To study the photochemical behavior of PAzo at the macroscale, the prealigned PAzo films were prepared by stretching to 200% strain, kept at 60 °C overnight, and followed by cooling to room temperature. This process produced alignment of the azobenzenes in the main chain of PAzo, thus maximizing the photochemical response.^47^Figure 4e shows that the surface temperature of the aligned PAzo film increased by 2 °C when irradiated by 470 nm light (0.2 W/cm^2^) for 60 s to reach a photostationary state, indicating that the photochemical reaction occurred (Figure 3d,f) with a negligible photothermal effect (Figure 3e,f) on the phase structure and thermal performance of the PAzo elastomer. In response to such stimulation, the prealigned PAzo film showed a clear drop of 0.25 MPa stress (Figure 4f), which may be attributed to the increased steric hindrance from the *trans*-to-cis photoisomerization of the azobenzenes in the PAzo film. As a result, photochemically induced expansion of the prealigned PAzo elastomer occurs. Notably, the photochemically induced deformation was less pronounced in the case of the cast PAzo film without prealignment due to its randomly oriented azobenzenes. By contrast, the surface temperatures of the PAzo films increased up to around 40 and 80 °C while increasing the irradiation intensity to 0.73 W/cm^2^ and 4.38 W/cm^2^ respectively (Figure 4e). In these two cases, apart from photochemical reactions, a pronounced photothermal effect occurs under light stimulation. This conclusion is supported by the observed thermal behavior of PAzo tested by DSC and DMA, showing the recrystallization and melting phase transition (*T*_m_ ∼ 38 °C) of PCL soft segments during the second heating (Figure 2c) and a marked decrease in the storage modulus with increasing temperature (from RT to 40 and 80 °C: Figure 2d). Therefore, the photochemical and photothermal coupling effects contribute to the overall mechanical response of PAzo to light stimulation. In fact, the observed photothermal effect is a common mechanism among described photoresponsive materials. The positive coefficient of thermal expansion (Figure S7) also demonstrates the contribution of the photothermal effect to photoinduced expansion. The thermal viscoelastic mechanical properties of the PAzo film can be quantified by DMA (Figure 2d). There is a relatively stable elastic region (*E*′ ≫ *E*″) with a flat valley of tan δ ≈ 0.18 after a glass transition temperature of up to 40 °C, with a slow decrease in the storage modulus of PAzo, followed by a dramatic decrease (from 40 to 80 °C) when the temperature reaches the *T*_m_ (∼38 °C) of PCL soft segments.

Dynamic tensile tests can capture the reversibility and viscoelastic behaviors of cast PAzo (without stretching and annealing treatments) caused by photochemical and/or photothermal effects. When the photoisomerization of azobenzenes (photochemical effect) of PAzo was greatest under 470 nm light stimulation with a low intensity of 0.20 W/cm^2^ (Figure 4g), the maximum cyclic tensile stress drops slightly by ∼0.1 MPa, and then almost fully recovers when the light source is switched off. We propose that this is due to the relatively short half-life of *cis*-to-*trans* relaxation^44^ of the azobenzene derivative (DDB) in PAzo at room temperature. On the other hand, coupling photochemical–thermal effects took place when the light intensity increased. Figure 4h demonstrates that the dynamic tensile stress decreased from ∼1.3 to ∼1.1 MPa when the light was on 0.73 W/cm^2^ and partially recovered to ∼1.2 MPa when the light was off. The decrease of ∼0.2 MPa resulted from the combination of photothermal and photochemical effects of PAzo. A more significant decrease in the dynamic tensile stress from ∼1.2 to ∼0.1 MPa occurred when the light was on 4.38 W/cm^2^ for 60 s and then recovered partially to ∼0.4 MPa (Figure 4i). Such pronounced stiffness softening is attributed to the predominant photothermal effect of PAzo when irradiated with high-intensity light. The high temperature (∼80 °C) of PAzo generated by high-intensity light was above the *T*_m_ of PCL soft domains, which induced the ordered or tight chain packing of PCL soft segments to relax into a complete amorphous rubbery phase, thus softening the material. It should be mentioned that a small fraction of the stress loss of PAzo appeared to be unrecoverable in the initial light stimulation (≤0.73 W/cm^2^, Figure 4g,h) and became pronounced with increasing light intensity (4.38 W/cm^2^, Figure 4i). However, these rapidly reached a new level of reversible stress under repeated-pulse radiation conditions. The unrecoverable part of the stress may be due to the viscoelastic behavior of the soft domains in the phase transition (*T*_m_ ≈38 °C, Figure 2c) of a small fraction of crystals or physical entanglement of PCL soft chain segments formed during the casting and stretching process (Figure 3). Nevertheless, PAzo retained its outstanding dynamic elasticity at the corresponding level once the *trans*–*cis* isomerization in the hard domains and phase changes in the soft domains stabilized during and after light irradiation (Figure 4h,i).

Dynamic mechanical mapping of PAzo film surface before and after light illumination (0.2 W/cm^2^) by AFM provides more information on the role of nanophase structure and their photomechanical responses (Figure 4j–m). Figure 4n–q shows the corresponding AFM histograms before and after light illumination. Figure 4j,n displays a flat surface (−375 to 375 pm) of the PAzo film with little height change after in situ light irradiation. The phase images show uniform nanophase separation between the hard domains (darker domains) and soft domains (bright domains) before and after light illumination. Meanwhile, there was a distinct peak phase shift from ∼72° to ∼75° (Figure 4o), and the boundary between the nanophases became less distinct following light stimulation (Figure 4k), suggesting a softening effect of the hard domains. Figure 4l,m maps the nanoviscoelastic properties (frequency and loss tangent, respectively) of PAzo before and after in situ light illumination. The frequency peak becomes more diffuse toward the lower range, although the peak frequency appears to increase slightly (Figure 4p). These frequency results are directly correlated to the surface stiffness and confirm that the hard domains become softer after illumination and that the interface between hard and soft domain becomes blurred. A slight increase in the loss tangent histograms (Figure 4q) echoes the same view. These nanoscale phase changes reveal an intrinsic correlation between the nanostructure and photochemical nanoviscoelasticity of PAzo, due to the *trans*-to-cis isomerization of the azobenzenes within the hard domains under low-intensity light irradiation. The photoresponsive mechanisms of the PAzo elastomer in response to either photochemical or photothermal effects as above are illustrated in Figure 3. By comparison, PCL–PUU did not show obvious phase or height changes after in situ light stimulation (Figure S8). All AFM images support our proposed mechanisms for the photochemical nanomechanics of PAzo and the correlation with its intrinsic nanophase separation structure after irradiation with 470 nm blue light at a low intensity. Due to the setup constraints, it is difficult to measure the dramatic surface softening and deformation in real-time at high light intensity (4.38 W/cm^2^) using an AFM cantilever. However, the considerable stress drop of PAzo at this higher intensity is evident in the dynamic tensile tests shown in Figure 4i, indicating pronounced viscoelastic behavior with a significant decrease in the storage modulus under 4.38 W/cm^2^ light illumination. It is also of note that less than half of the stress was recovered when the light was removed, implying slow packing and recrystallization of PCL soft domains, which could not recover quickly as the temperature fell quickly.

These results demonstrate the photodriven stiffness softening effect of PAzo under light with is triggered by a photochemical and photothermal coupling effect depending on the light intensity, observed in both DMA and AFM tests (Figure 4g–q). Photoinduced softening results from photochemical effects of low-intensity light (Figure 4f,g), coupled with photothermal effects as the intensity increases (Figure S7). The photoinduced expansion deformation of pure PAzo films under light depends on molecular chain conformation, alignment of azobenzene-containing rigid chain segments, and intensity of the light, regardless of the light wavelength chosen. Photochemical and photothermal coupling predominates when the light intensity is ≥0.73 W/cm^2^. It should be noted that the photoswitched glass transition from the azobenzene-containing hard domains of PAzo was not as pronounced as that reported for the side chain and thermoset azopolymers,^51,52^ which may be related to its unique nanophase structure with uniform nanoisland-like hard domains (glass phase) among the continuous soft domains (rubber phase, Figure 3). In this case, the photothermal effect is predominantly contributed from the chain relaxation of the soft domains (*T*_m_), which is revealed by DSC or DMA (Figure 2c,d), while the *T*_g_ of the hard domains is too high to be detected (>thermal degradation temperature, 250 °C, see TGA in Figure S2). This mechanism of photodriven stiffness softening is reflected in driving the bending behavior of PAzo-containing actuators below.

### 2.3 Two Bending Modes of Bilayer Actuators under LED Illumination with Varying Light Intensities

**Figure 5:**
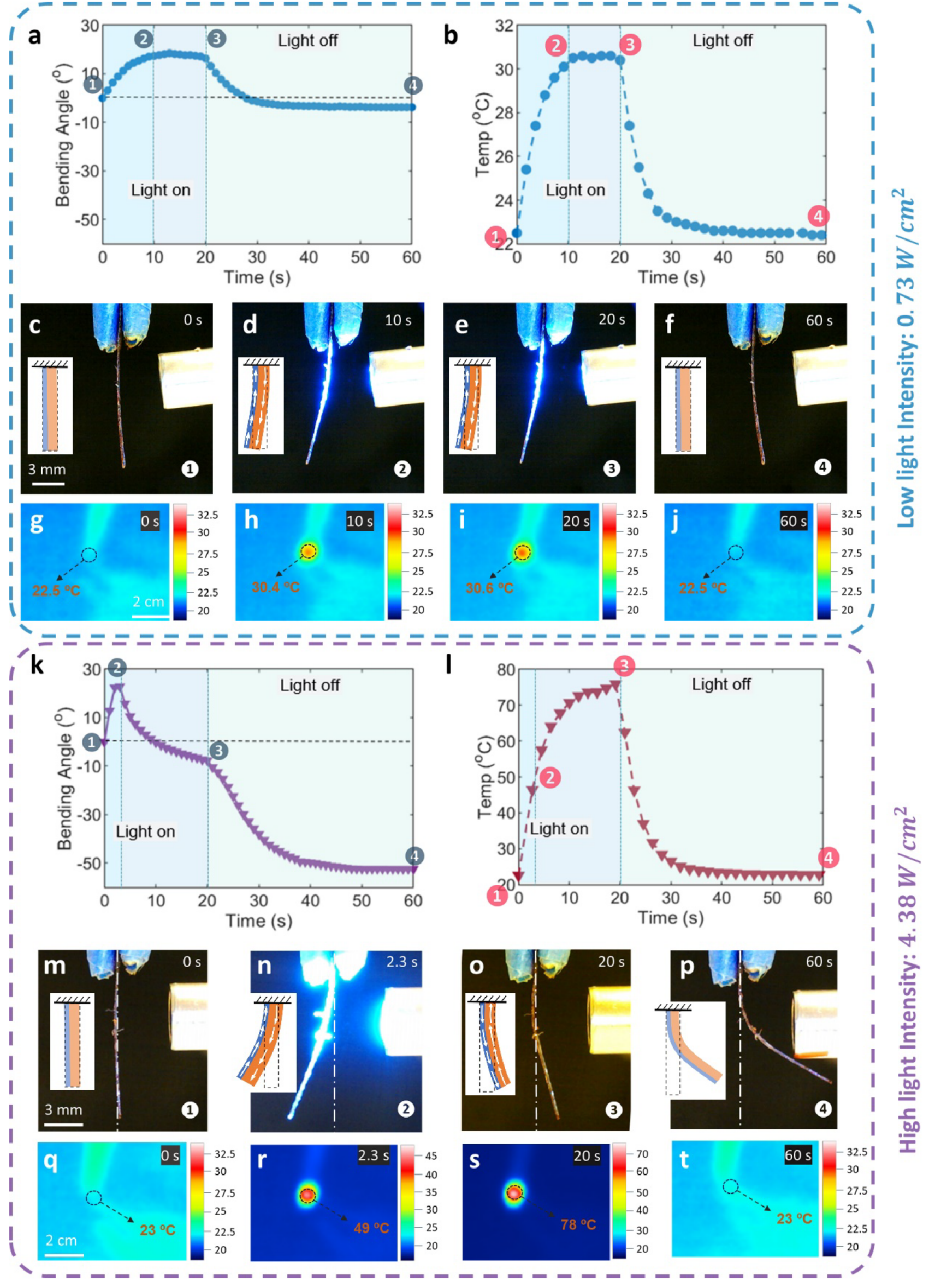
Two bending modes of the PAzo/Kap bilayer actuator (10 × 2 × 0.16 mm^3^) under 470 nm irradiation with different light intensities of 0.73 W/cm^2^ (low) and 4.38 W/cm^2^ (high). Low light intensity: (a) bending angle as a function of time as light was turned on (20 s) and off (40 s) on the PAzo/Kap film side; (b) related surface temperature of PAzo/Kap film was plotted over time during the irradiation period; (c–f) digital images and corresponding stress distribution (yellow layer: PAzo, blue layer: Kapton); (g–j) infrared images of the bending process of PAzo/Kap actuator during one cycle of irradiation. High light intensity: (k) bending angle as a function of time and corresponding stress distribution of the biolayer actuator when light was turned on (20 s) and off (40 s) on PAzo/Kap film; (l) related surface temperature of PAzo/Kap film was plotted over time during the irradiation period; (m–p) digital images and corresponding stress distribution of the biolayer actuator (o: the threshold moment when the light is turned off); (q–t) infrared images of the bending process of PAzo/Kap during one cycle of irradiation.

A visible-light-driven actuator with a bilayer structure that combines PAzo and Kapton sheets was designed and fabricated. As shown in Figure S9, PAzo has a Young’s modulus of around 17.6 MPa which is much lower than that of the commercial Kapton film (889.9 MPa) and is complementary with a higher stretchability with an ultimate strain up to 575.2%, compared to 20.7% for Kapton. The stress strain behaviors suggest that, unlike Kapton, PAzo inherits the characteristics of a poly(urethane urea) elastomer, which is highly flexible and stretchable due to its outstanding entropic elasticity. Under exposure to 470 nm light at different intensities, the PAzo/Kap bilayer structure exhibited two bending modes. For mode I (Figure 5a–j), upon light irradiation with a low intensity of 0.73 W/cm^2^, the bilayer film bent away from the light source and then recovered to more or less the original position when the light was off. For mode II, as shown in Figure 5k–t, upon light irradiation with a high intensity of 4.38 W/cm^2^, the actuator bent away from the light source (①-②) and then recovered to the zero state or even reversely bent toward the light source during the period with light on (②-③), with a consecutive bending further toward the light source even after the light was off (③-④).

The actuation mechanisms of these two modes can be explained based on the photothermal and photochemical coupling mechanism under light irradiation with an intensity ≥0.73 W/cm^2^, including the photothermally induced phase transition of soft domains of PAzo revealed above. In a typical photoactive bimorph actuator, one layer is photoactive, i.e., the PAzo layer in this case, has a photochemical/photothermal coupling effects, leading to a volume/shape change (to expand or shrink) under light stimulation; another layer is a passive structural layer, Kapton sheet, which is not sensitive to light irradiation and remains stable in the range of temperature changes. Therefore, the active layer dominates the volume/shape change but is simultaneously constrained by the passive layer. The competition between layers thus built up the tension or compression stress field across the thickness of each layer of the actuator (as shown by the diagram insets of the stress distribution in Figure 5c–f, m–p), resulting in different bending behaviors under light stimulation.^67^

More specifically, in the case of bending mode I, as light was on from ① to ② and ③ (Figure 5c–e), the temperature increased gradually from 22.5 to 30.4 °C and remained at around 30.4 °C (Figure 5g–i. Both PAzo and Kapton were proven to contribute to the temperature increase, as demonstrated by large changes in temperature during and after light irradiation (Figure S10). By contrast, the temperature of the control group, PCL–PUU (without bonding with azobenzene), changed little under the same light illumination. Through the strain-temperature sweep by DMA (Figure S7), the coefficient of thermal expansion (CTE) of PAzo is calculated to be 204.4 × 10^–6^ °C^–1^, ten times greater than that of Kapton (20 × 10^–6^ °C^–1^, as listed in Table S4). Therefore, under the same temperature change, the photothermal expansion of PAzo is already much greater than that of Kapton, plus a photochemical softening effect, resulting in the actuator bending away from the light source with tension stress generated within the PAzo layer and compression stress within the Kapton layer, as illustrated in Figure 5d,e. When the light was turned off, the specimen cooled to room temperature from ③ to ④ (Figure 5b,i,j); hence, the bilayer actuator simultaneously recovered to its original position (Figure 5f). For bending mode II, as light was on from ① to ② and ③ as shown in Figure 5m–o, the temperature increased from 23 to 49 and 78 °C (Figure 5q–s), which was above the *T*_m_ of PAzo soft domain (38 °C) (Figure 4b); at the same time, the storage modulus of PAzo decreased significantly (Figure 2c). As a result, the crystalline packing in soft domains of PAzo melted and became a completely soft rubbery phase, contributing to the dramatic decrease in the stiffness of PAzo, in addition to the photochemical softening effect in glassy hard domains. Consequently, the bending energy in the Kapton film was released from ② to ③ (Figure 5n,o). As a result, the bilayer film bent away from the light source first under tensile stress within the PAzo layer and under compression stress within Kapton, then recovered to the original position, and even bent reversely toward the light source (Figure 5m–o). After the light was turned off from ③ to ④, the temperature of the specimen dropped dramatically (Figure 5s,t) and the volume of the PAzo layer decreased during cooling, resulting in the bilayer actuator consecutively bending toward the light source under compression stress within the PAzo layer and tensile stress within Kapton (Figure 5o,p). Positioning the PAzo layer away from the light source is likely to influence both the bending speed and the maximum bending angle of the bilayer actuator, primarily for two reasons. First, the presence of the Kapton film can reduce the light intensity, consequently reducing the photochemical-induced expansion of the PAzo film. Second, the different thermal expansion coefficients of Kapton and PAzo films (Table S4) contribute to a notable deceleration in the photothermal-induced expansion of the PAzo film.

The above results demonstrated that these two bending behaviors were driven by photothermal and photochemical coupling effects, including photothermal-induced phase transitions and resulting changes in nanophase structure of the polymer under high-intensity light in the case of mode II. Nevertheless, the intrinsic correlation between the deflection angle and the dual-photoeffects of such a responsive elastomer has yet to be explored. Hence, we performed a theoretical analysis of the bending behavior of the bilayer structure based on Timoshenko’s theory for bimetal thermostats, with the assumption of linear elastic mechanics that only photothermal effect occurs^68^ (details in the Supporting Information). Interestingly, the theoretically calculated angle (6.14°) is much lower than that observed by experimental measurement (∼20°), which suggests that the photothermal mechanism may not be the predominant contributor to the bending behavior upon low-intensity light irradiation. In other words, it is speculated that the photochemical mechanism, cis-to-*trans* isomerization of azobenzenes, intrinsic entropic elasticity of the PAzo elastomer, and viscoelasticity owing to photothermal-induced phase transitions play more essential roles in the resulting large bending angle, contributing to about 69.32% of the deflection angle in total.

### 2.4 Tunable Actuation Performance of the PAzo Based Bilayer Actuator

**Figure 6:**
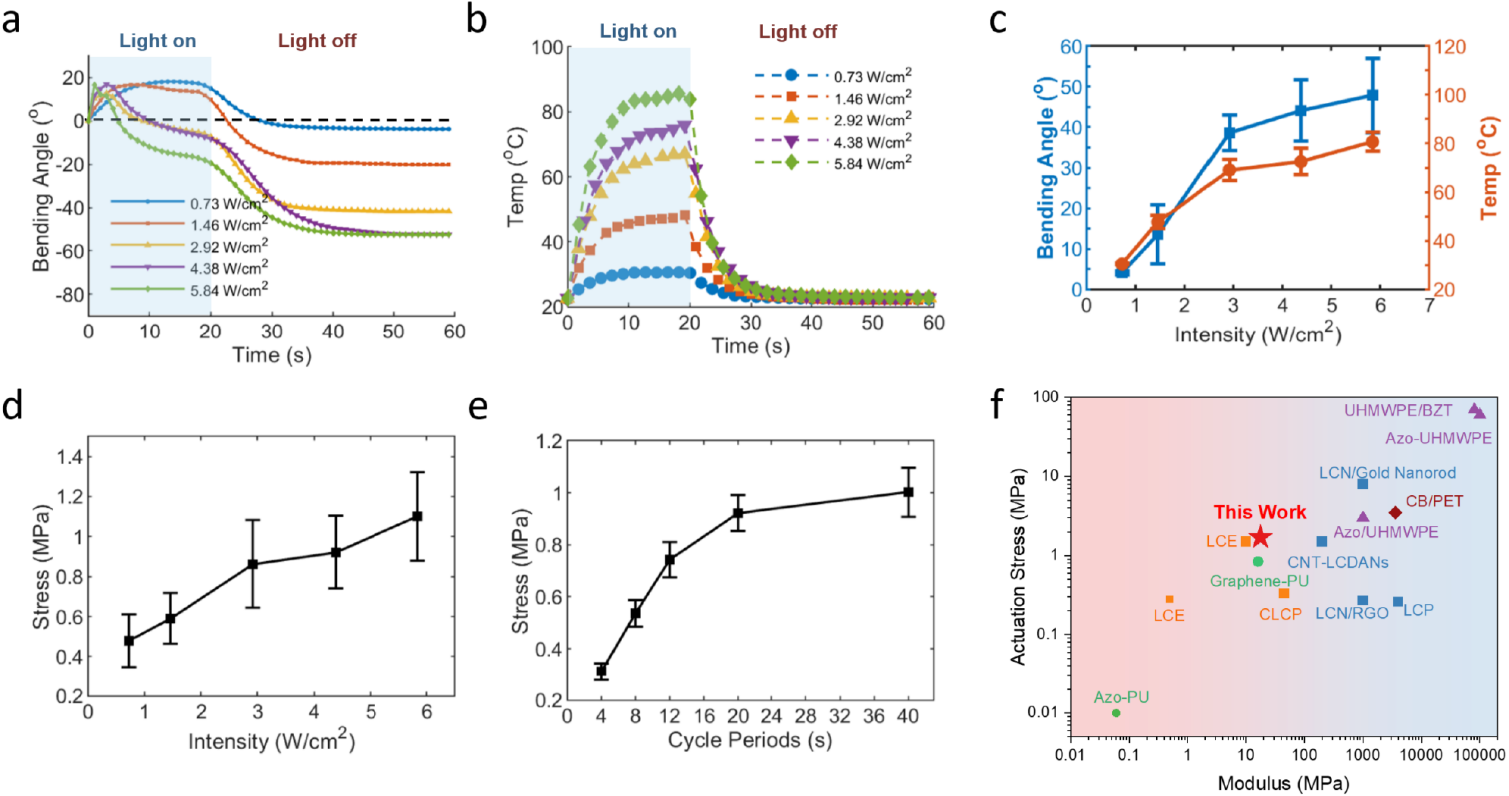
Bending behavior and actuation stress of PAzo/Kap bilayer actuator (10 × 2 × 0.16 mm^3^) by 470 nm light irradiation. (a) Bending angle changes of the bilayer actuator exposed to 470 nm LED light with different light intensities; (b) light-induced temperature increase of the PAzo/Kap film at different light intensities; (c) absolute residual bending angles after light irradiation and the highest temperatures upon light irradiation with different light intensities; (d) actuation stress of the bilayer versus light intensity with a cycle period of 40s (light on 20s and off 20s); (e) actuation stress versus cycle periods under irradiation with an intensity of 4.38 W/cm^2^; (f) Ashby plot of actuation stress versus modulus of PAzo and other photoresponsive materials/actuators including Azo-PU,^47^ Graphene-PU,^55^ LCE,^6,59^ CLCP,^61^ CNT-LCDANs,^60^ LCN/RGO,^15^ LCP,^14^ LCN/Gold Nanorod,^13^ Azo/UHMWPE,^69^ UHMWPE/BZT,^64^ and CB/PET.^9^

The light-driven actuation function of the PAzo-based bilayer actuator was systematically quantified under different radiation conditions. Figure 6a demonstrates the changes in the bending angles under different light intensities. The direction in which the specimen bends away from the light source is defined as the positive direction (hence a positive bending angle), while the direction in which the specimen bends toward the light source is defined as the negative direction. Figure 6b shows the corresponding temperature changes of the actuator during the irradiation period with different light intensities. Figure 6c summarizes the final absolute bending angles after one cycle of irradiation and the maximum temperatures of the actuator during the irradiation period. With increasing light intensity (below 6 W/cm^2^), the bending angle increased from 5° to 50°, and the temperatures rose from 30 to 80 °C.

To analyze the photomechanics of the bilayer actuation, both ends of the actuator were clamped in an Instron tensile tester, with 1% prestrain applied to the bilayer under relaxation mode to record the stress changes during periodic light irradiation. The dimensions of the actuator are 10 × 2 × 0.16 mm^3^ (the thickness of Kapton is a constant of 0.05 mm for all PAzo/Kap actuators), the light intensity is 4.38 W/cm^2^ and the cycle period is 40s (20s on and 20s off). Figures 6d and S11a show that the actuation stress increases with increasing light intensity from 0.73 W/cm^2^ to 5.84 W/cm^2^, consistent with the trend of the bending angle in Figures 6c and S11c. As expected, a large bending angle and a high force are generated by high-intensity light. The time–temperature dependence of the stress generated is also evident in the initial increase in the actuation stress and then gradually reaches a plateau of about 1 MPa as the on-and-off cycle period of stimulation increases (Figures 6e and S12b). With increasing the thickness of the PAzo/Kap bilayer film from 0.10 to 0.25 mm (Kapton was maintained at 0.05 mm), the actuation stress decreased as shown in Figure S12c,d, which was attributed to an exponential increase in the bending stiffness of the actuator (at a power of 3 of the thickness). All these results suggest that the actuation performance of the PAzo/Kap actuator could be well designed and controlled by a range of the input light intensity, cycle period, and geometry of PAzo. Figure S13 demonstrates the force output stability of the bilayer actuator after 100 on/off illumination cycles of 470 nm LED light. Besides, the output stress or force of the PAzo/Kapton actuator is comparable to, or even outperforms, some of the light-driven polymers and actuators reported in the literature, such as LCN/Kap (1.58 MPa),^7^ UHMW-PE (3 MPa),^69^ LCP (0.35 MPa),^61^ and ELCN/Kap (0.4 MPa).^63^ The Ashby plot in Figure 6f compares the actuation stress and modulus of PAzo with those of other photoresponsive materials/actuators reported in the literature.

### 2.5 Light-Driven PAzo Based Robotic Fingers for Playing Piano

**Figure 7:**
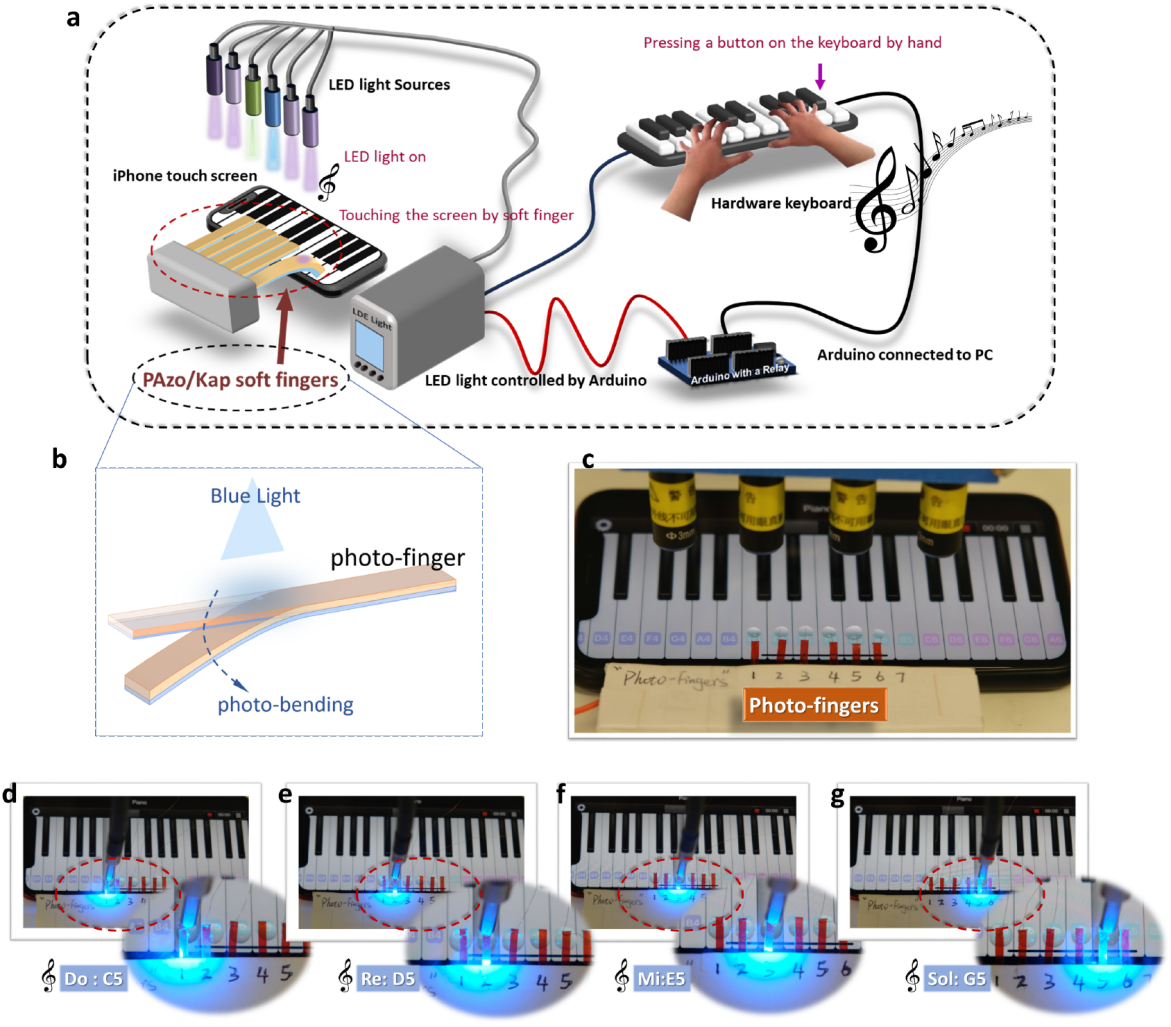
Design, fabrication, integration and operation of light-driven soft robotic fingers to play a piano. (a) Schematic illustration of the working principle of playing a piano application on a smartphone by soft touch robotic fingers based on customized light stimulator hardware controlled by human fingers. (b) Enlarged illustration of “photofinger” (c) Soft robotic fingers mounted above the touch screen with a piano app turned on; (d-g) Snap shots of different soft robotic fingers playing the notes from “Mary had a little lamp” song (470 nm LED with a light intensity of 4.38 W/cm^2^).

Inspired by an electro-ionic soft actuator controlled by electricity for playing an electronic piano,^70^ we demonstrated untethered photoresponsive soft robotic “fingers” (PAzo/Kap bilayer actuators) to play a keyboard piano on an iPhone touch screen by remote light (Figure 7). As shown in the illustration of Figure 7a, the robotic strip “fingers” were made from the PAzo/Kap bilayer actuators (“photofinger” shown in Figure 7b), which are able to bend to touch the iPhone screen keyboard of a piano app to play music by light stimulation. The LED light was controlled by a hardware keyboard designed and made in-house from micro switches (Figure S16a) controlled by an Arduino with a relay. Figures 7c and S16b display the digital images of the different LED heads for controlling each PAzo/Kap strip finger above the digital keyboard on the iPhone screen and the integrated electronic components in this application. The snap shots in Figure 7d–g demonstrate the soft robotic fingers playing the notes from the “Mary had a little lamp” song. Video 4 demonstrates the whole process of playing this song. To enhance the bending speed, we used a light intensity of 4.38 W/cm^2^, where the photothermal effect predominates. This demo shows that our PAzo/Kap actuation is controllable for sophisticated performance by a light stimulus.

## 3 Conclusions

A new visible light-responsive and highly compliant elastomer (PAzo) has been successfully synthesized via a facile route by introducing covalently bonded photoresponsive molecules (i.e., azobenzene derivatives) into the main chain of biocompatible poly(urethane urea). PAzo elastomer shows pronounced and robust stiffness softening under blue light stimulation while possessing outstanding hyperelasticity and biocompatibility owing to its azobenzene photoresponsive function as well as intrinsic nanophase structure through self-assembly of soft and hard segments of block copolymeric chains. A light-driven bilayer actuator made from PAzo-Kapton demonstrated two distinct bending modes under different light intensities, providing enormous potential in the design and programming of actuators for various actuations and motions. The unique actuation mechanisms driven by tunable photochemical and photothermal coupling effects have been thoroughly studied experimentally and theoretically. Based on the PAzo/Kap bilayer actuator, the application of light controlled soft robotic fingers for playing piano on a smartphone was demonstrated. The understanding of photostiffness softening mechanism driven by photothermal and photochemical couple effects in response to the chain relaxation of soft and hard segments of linear chain polyurethane elastomer contributes to the fundamental knowledge of light responsive materials. The PAzo elastomer and its light-driven stiffness softening features provide a robust flexible material, principle, and scalable approach for the design and manufacture of light-driven wearable/implantable actuators and soft robots for the restoration of physiological function or assistive devices for medical rehabilitation.

## 4 Experimental Section

### 4.1 Materials

Polycaprolactone diol (PCL) (Mn = 2000), 4,4′-methylenebis(cyclohexyl isocyanate) (HMDI) (90%), and disperse diazo black 3BF (DDB) were purchased from Fisher Scientific UK Ltd. and used as received. The bismuth neodecanoate (BN) catalyst, ethylenediamine (≥99.5%) chain extender, anhydrous dimethylacetamide (DMAc) (99.8%), and 1-butanol (99.8%) solvents were purchased from Sigma-Aldrich and used without further purification. Commercial Kapton polyimide tape was purchased from RS Components UK.

### 4.2 Synthesis of PAzo

Polycaprolactone diol (14.48 g, 7.24 mmol) and 4,4′-methylenebis(cyclohexyl isocyanate) (6.80 g, 25.92 mmol) were dissolved in 30 mL of anhydrous dimethylacetamide in a 250 mL three-neck flask under nitrogen. After the solution was degassed by bubbling nitrogen for 0.5 h, bismuth neodecanoate (0.26 g, 0.36 mmol) was added. The reactants were then heated at 80 °C for 6 h to form a solution of the prepolymer. After the solution cooled to room temperature, disperse diazo black 3BF (DDB, 1.99 g, 7.76 mmol) in 80 mL of anhydrous and degassed dimethylacetamide was added dropwise to react with the prepolymer solution under vigorous stirring. The reaction was continued at 60 °C for 6 h and then cooled to 40 °C. The final step of the chain extension reaction was completed by adding ethylenediamine (0.66 g, 10.92 mmol) in 20 mL of dimethylacetamide dropwise to the reaction solution under vigorous stirring for about 55 min. After completion of the polymerization, 1-butanol (1.00 g, 13.49 mmol) was quickly added to the solution and stirred for over 1 h. The polymer solution was then poured into a mold and dried into a film in a 60 °C oven for 24 h before post processing.

### 4.3 Physiochemical Characterization

A series of advanced scientific instruments were used to characterize the synthetic materials.

#### 4.3.1 Uniaxial Tensile Testing

Samples in dumbbell shape (*n* = 5, length of 10 mm, width of 2 mm) were subjected to uniaxial loads at 10 mm/min using an Instron 5565 tester (Instron Ltd., Norwood, MA, USA) with a 50 N load cell. Nominal stress–strain curves of the samples were plotted to calculate the ultimate tensile strength, strain at break, tensile modulus, and toughness.

#### 4.3.2 UV–Visible Spectrophotometer (UV–Vis)

UV–vis absorption spectra of PAzo-DMAc solutions were recorded by a Jasco V-630 UV–visible spectrophotometer (Tokyo, Japan) on 1 cm path length quartz cells at 300–600 nm and a scanning rate of 400 nm/min to detect the photoisomerization properties of PAzo before and after 365 and 470 nm light irradiation.

#### 4.3.3 Differential Scanning Calorimetry (DSC)

DSC measurements were performed on a TA Instrument Q2000 using an aluminum hermetic crucible. PAzo film samples were heated and cooled at a constant rate of 10 °C min^–1^ between −80 and 220 °C under a nitrogen atmosphere for three cycles. The phase transition temperatures of the PAzo films were determined from the second scan.

#### 4.3.4 Attenuated Total Reflectance Fourier Transform Infrared Spectroscopy (ATR-FTIR)

A FTIR spectrophotometer was used to analyze the chemical group of the PAzo films using a Jasco FT/IR-4200 Spectrometer (JASCO Inc., USA). Spectra were recorded in the wavenumber range of 4000–500 cm^–1^ by the accumulation of 20 scans.

#### 4.3.5 Dynamic Mechanical Analysis (DMA)

The viscoelastic responses of PAzo were determined using a DMA Q800 instrument (TA Instruments). The PAzo (a 18.81 × 7.60 × 0.31mm^3^ rectangular film) was loaded with a dynamic tensile strain of 1% at a frequency of 1 Hz and the temperature was increased at a rate of 2 °C/min from −70 to 100 °C.

#### 4.3.6 Wide-Angle X-Ray Diffraction (WAXD)

The phase structure of PAzo was examined by a WAXD instrument (Bruker D8 Advance, Germany).

#### 4.3.7 Universal Measurement Spectrophotometer (UMS)

The UV–vis/NIR transmission of the PAzo film was measured using a Cary 7000 Universal Measurement Spectrophotometer (UMS, Agilent Technologies, USA) across the spectral range of 250 to 2500 nm.

#### 4.3.8 Wide/Small Angle X-Ray Scattering (WAXS/SAXS)

The X-ray scattering patterns of the samples were further examined by using a WAXS/SAXS instrument (Ganesha 300XL, SAXSLAB, Denmark). The PAzo film samples were firmly attached to the sample holder and placed perpendicular to the beam. All of the X-ray scattering measurements were carried out using a 2 mm beam stop under vacuum, at room temperature, with a wavelength of 1.5418 Å (Cu-source). Silver behenate (peak at 0.1076 Å^–1^) was used for calibration before sample scanning. For the middle angle X-ray scattering (MAXS) ranging from 0.015 to 0.65 Å^–1^, the beam size at the sample position was about 0.4 × 0.4 mm^2^, and the distance from the sample to the detector is about 441 mm. For the extra small angle X-ray scattering (ESAXS) ranging from 0.0035 to 0.18 Å^–1^, the beam size at the sample position was about 0.2 × 0.2 mm^2^, and the distance from the sample to the detector was about 1491 mm. The characteristic separation distance (*d*-spacing) was calculated using Bragg’s law *d* = 2π/*q*, where *q* is the scattering vector.

#### 4.3.9 Photochemical Performance of the PAzo Film

To evaluate the photochemical actuation performance of the films upon blue light exposure (470 nm, 226 mW/cm^2^), the films (stretched to 200% strain, annealed at 60 °C overnight in an oven and then cooled to room temperature, 20 × 5 × 0.15 mm^3^), were clamped in an Instron 5565. A prestrain of 1% was set at the tensile tester under the stress relaxation mode for each sample. Force values were changed under light irradiation and recorded by the Instron.

#### 4.3.10 Atomic Force Microscopy (AFM)

AFM scanning was performed at room temperature using an MFP-3D system (Asylum Research, USA) in tapping mode with a cantilever (*k*: 8.45–37.97 N/m) at a scan frequency of 1 Hz. The sample was prepared by drop-casting the PAzo and PCL–PUU solutions (∼2 wt %) on a silicon wafer. Several specimens were scanned in different regions to ensure reproducibility of the results.

### 4.4 Preparation of the Light-Driven Bilayer Actuator

A PAzo in DMAc solution with 18% concentration was prepared and used for fabrication of the actuators and films. For the actuators, 10 mL of the PAzo/DMAc solution was poured onto a circular glass Petri dish (diameter of 10 cm) with a Kapton film (thickness of 0.05 mm) stuck on it, using a syringe. The Petri dish was then placed in a vented oven at 60 °C for 24 h. After evaporation of the DMAc, 2D-cast PAzo-Kapton bilayer actuators were peeled off the master surface. The thickness of the actuator could be controlled by adjusting the volume of the PAzo/DMAc solution. For the cured films, the PAzo solution was poured into a glass mold with Kapton tape and cured in a 60 °C vacuum oven overnight. The fully cured film was then peeled off and cut into various shapes.

### 4.5 Characterization of the Performances of the Light-Driven Soft Actuators

The photomechanical behavior of the samples was investigated by irradiating the films with a 470 nm LED (CoolLED pE100, UK) and a 365 nm UV-LED (TAOYUAN, H.K.) with adjustable light intensity. As shown in Figure S14a, the PAzo/Kap bilayer is clamped at one end as a cantilever actuator and irradiated by 470 nm blue light from the PAzo side. The dimensions of the bilayer actuator with dimensions of rectangular length, width, and thickness are 10 × 2 × 0.16 mm^3^ (with the thickness of Kapton being constant at 0.05 mm), and the light intensity is set to 0.73 W/cm^2^ for low intensity and 4.38 W/cm^2^ for high intensity, with the irradiation area at the top half of the actuator unless otherwise stated. We adjusted the light intensity to actuate the bilayer while recording the bending angle and temperature changes in real-time using video. The intensity of UV exposure was measured using a UV radiometer (CON-TROL-CURE, Silver Line). A USB digital microscope (20–200 Magnification, RS Component, UK) was used to record the bending behavior of the PAzo/Kap actuator under light irradiation. Infrared videos were taken with an infrared fusion technology camera (Testo 885, German) to capture thermal images and videos of the illuminated films. Thermographic data acquisition was carried out through the IRSoft2 application associated with the IR camera. The moving velocity was obtained by analyzing the captured videos using Tracker software.

To evaluate the actuation performance of the films upon UV or blue light exposure, the films (the same size as the dumbbell samples used in the tensile test) were clamped in an Instron 5565 instrument. A prestrain of 1% was set at the tensile tester under the stress relaxation mode for each sample. Force values were changed under light irradiation and recorded by the Instron.

### 4.6 Fabrication and Operation of Light-Driven Soft Robotic Fingers for Playing Piano

To demonstrate the application of soft robotic fingers for playing piano, micro switches (RS Component, UK) were used for fabricating hardware keyboards, and an Arduino UNO (RS Component, UK) was utilized for developing the circuit of this application. The integration of the whole circuit is shown in S16.

## Supporting Information Available

Synthesis of PCL–PUU (PAzo without Azo content), thermogravimetric analyses (TGA) of PAzo, differential scanning calorimetry (DSC) curves of PAzo film, wide/small-angle X-ray scattering (WAXS/SAXS) of PAzo; comparison of the mechanical performances between PAzo and Kapton characterized via tensile tests; comparison of the temperature changes among PAzo, PCL–PUU, and Kapton when irradiated by 470 nm LED light with different light intensities; theoretical analysis of the bending angle of the bilayer actuator upon light irradiation with low intensity; coefficient of thermal expansion (CTE) of PAzo determined by thermal contraction/expansion experiments; energy conversion of the PAzo/Kap bilayer actuators; force output stability of the bilayer actuator after 100 light on–off cycles, storage modulus of PAzo at different temperatures, and optical imaging of the control system for the application of soft robotic fingers to play a piano app; comparisons of light-responsive materials reported in the literature (Table S1); thermal properties of PAzo before and after light irradiation (Table S2); *d*_spacing_ of PAzo calculated from the WAXD and SAXS results (Table S3); material properties of PAzo and Kapton for theoretical calculations (Table S4) (PDF)
Bending mode I of the PAzo/Kap bilayer actuator under 470 nm light irradiation at low intensity (Movie 1) (MP4)
Bending mode II of the PAzo/Kap bilayer actuator under 470 nm light irradiation at high intensity (Movie 2) (MP4)
Keyboard test of light-driven robotic fingers for playing a piano app (Movie 3) (MP4)
Light-driven robotic fingers playing “Mary had a little lamp” on a smartphone piano app (Movie 4) (MP4)
