# Supplementary material for: Tunable Light-Responsive Polyurethane-urea Elastomer Driven by Photochemical and Photothermal Coupling Mechanism
Source: ACS Appl Mater Interfaces. 2024 Apr 6;16(15):19480–95. doi: 10.1021/acsami.4c00486 (PMC11040532; doi:10.1021/acsami.4c00486)
Supplement: Supplementary file 1 — am4c00486_si_001.pdf [file am4c00486_si_001.pdf]

Supporting information

Tuneable Light-Responsive Polyurethane-urea Elastomer Driven by Photochemical and Photothermal Coupling Mechanism

Lei Wu[1], Xia Huang[1], Meng Wang[1], Jishizhan Chen[1], Jinke Chang[1], Han Zhang[2], Xuetong Zhang[1,3], Andrew Conn[4], Jonathan Rossiter[4], Martin Birchall[5], Wenhui Song[1] *

[1]Centre of Biomaterials for in Surgical Reconstruction and Regeneration, Department of Surgical Biotechnology, Division of Surgery & Interventional Science, University College London NW3 2PF, United Kingdom

[2]School of Engineering and Materials Science, Queen Mary University of London, London E1 4NS, United Kingdom

[3]Suzhou Institute of Nano-tech and Nano-bionics, Chinese Academy of Sciences, Suzhou 215123, P. R. China

[4]Dept of Engineering Mathematics and Bristol Robotics Laboratory, University of Bristol Bristol BS8 1UB, United Kingdom

[5]UCL Ear Institute, Royal National Ear Nose and Throat and Eastman Dental Hospitals (UCLH NHS Foundation Trust) and University College London, London WC1X 8EE, United Kingdom.

Corresponding author. Email: w.song@ucl.ac.uk

Keywords: Photo-responsive elastomer, photochemical stiffness softening, photothermal stiffness softening, nanophase separation, light-driven soft robotics, untethered bionic fingers, polyurethane elastomer actuator

Synthesis of PCL-PUU (PAzo without Azo content)

The syntheses of PAzo and PCL-PUU were adapted from previous work[1]. In brief, Polycaprolactone diol (14.48 g, 7.24 mmol) and 4,4′-Methylenebis(cyclohexyl isocyanate) (6.80 g, 25.92 mmol) were dissolved in 30 mL of anhydrous dimethylacetamide in a 250 mL three-neck flask under nitrogen. After the solution was degassed with nitrogen for 0.5 h, bismuth neodecanoate (0.26 g, 0.36 mmol) was added. The reactant was then heated to 80 ℃ for 6 h to form a solution of the prepolymer. After the mixture was cooled down to 40 ℃, ethylenediamine (1.12 g, 13.49 mmol) in 100 mL of dimethylacetamide was added dropwise under vigorous stirring for about 1 h. Upon completion of the polymerization, 1-butanol (1.00 g, 13.49 mmol) was added for reaction termination. After the polymer solution was then stirred for 1 more hour and cooled to room temperature before being transferred to a 500 ml plastic screw top bottle. The resulting polymer solution was poured into a mould and cured in a 60 °C vacuum oven for 24 h before further processing.

In vitro experiments

Cell proliferation and viability Mouse embryonic dermal fibroblasts (NIH/3T3 cells, ATCC) were cultured in growth medium, which consisted of high glucose (4.5 g/L) Dulbecco's modified Eagles medium (DMEM) supplemented with 10% foetal bovine serum (FBS) and 1% penicillin/streptomycin, and incubated at 37 ℃ in a humidified atmosphere containing 5% CO_2. PAzo film was cut into discs (10 mm diameter and 0.2 mm thickness, n = 8 per group) and sterilised with 5% v/v hydrogen peroxide for 2 hours, then washed three times with sterile Phosphate Buffered Saline (PBS) for 5 minutes each time, and air-dried in a class II laminar flow hood. Coverslips were used as the positive control and underwent the same sterilisation procedure. Under sterile conditions, PAzo discs and coverslips were placed in non-adherent

sterile Falcon™ 48-well plates (Corning, 734-0956), and incubated with 500 μL of the growth medium for 24 h prior to cell seeding.

Scaffolds were seeded with six-passage (P6) cells at a density of 1×10^4 cells/scaffold in 500 μL of cell growth medium in 48 well plates. Fresh medium was replenished every other day, and the metabolic activity was evaluated by the PrestoBlue™ assay (Thermo Fisher Scientific, USA) on day 1, 3, 7, and 10, following the manufacturer's instructions, and standardised by total DNA content. Total-DNA content was quantified at the same time points as above using fluorescent Hoechst 33258 stain (Sigma-Aldrich, UK) according to the manufacturer's instructions.

Cell morphology imaging via confocal microscopy The morphology of 3T3 fibroblast was imaged utilising Leica TSC SP8 upright confocal microscope (Leica, Germany). Briefly, cell-laden scaffolds (n = 2 per group) were harvested at each day point, fixed with 4% paraformaldehyde (PFA; Sigma-Aldrich, UK) in PBS for 30 min at room temperature, and rinsed with PBS twice. Then, scaffolds were permeabilised with 0.1% Triton-X 100 (Sigma-Aldrich, UK) for 5 min and rinsed with PBS twice, then blocked with 1% bovine serum albumin (BSA) in PBS solution for 30 min. Following further rinsing, cells were stained with Alexa Fluor™ 488 Phalloidin (Thermo Fisher Scientific, USA) overnight for F-actin and DAPI solution (Thermo Fisher Scientific, USA) 15 min for nuclei and then kept in PBS before imaging. When imaging, samples were taken out from PBS and placed on a slide, covered with a glass coverslip to ensure flatness. Images were taken using the Leica TCS SP8 upright confocal microscope, with LASX software, 10 × and 20 × objective dry lens. To avoid sample dehydration, imaging process was limited to 5 min for each sample.

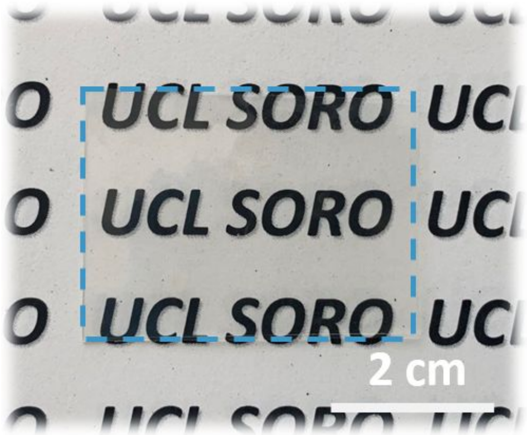

Figure S1. Optical image of PCL-PUU shown in the blue dashed box.

Thermogravimetric analyses (TGA) of PAzo

TGA were performed on a NETZSCH-TG 209 F1 Libra High Resolution TGA instrument by heating the samples up to 700 °C at 10 °C min^{-1} under N_2 atmosphere.

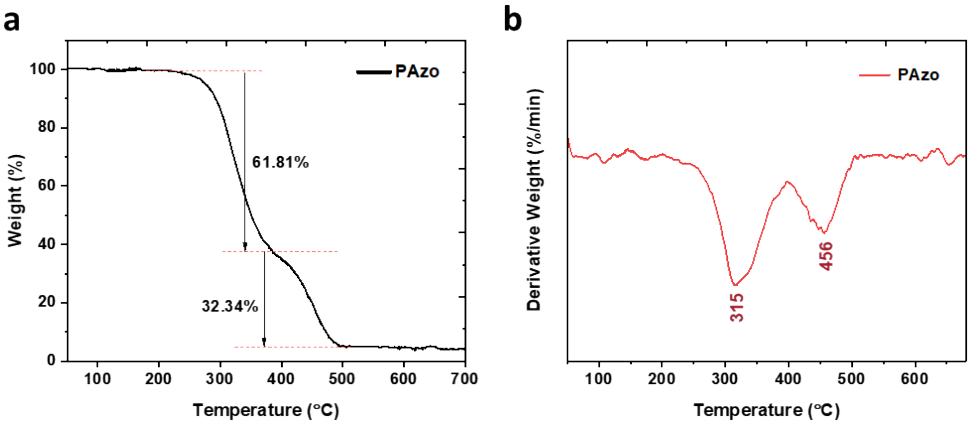

Figure S2. TGA curves of PAzo (a) Weight plotted with temperature; (b) Derivative weight plotted with temperature.

Differential Scanning Calorimetry (DSC) curves of PAzo film

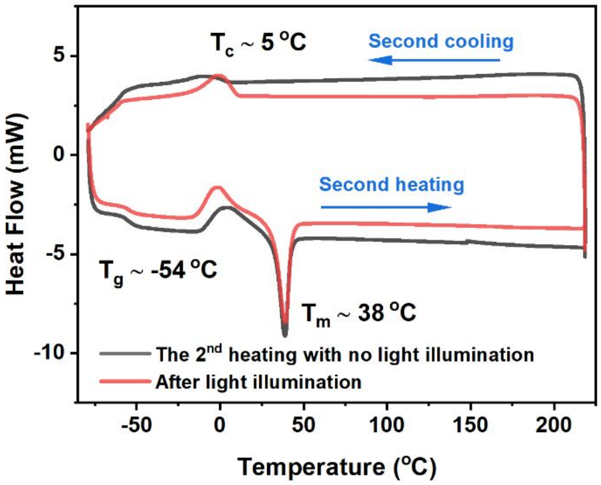

Figure S3. DSC scans of PAzo film without and with light illumination treatments (~1 W/cm^2 for 1 min) in the range from -80 ℃ to 220 ℃ (melting temperature of around 38 ℃), The recrystallisation and melting occurred are mainly due to DSC cooling or heating conditions which do not represent the actual crystallinity of the PAzo cast films.

Biological performance of PAzo film

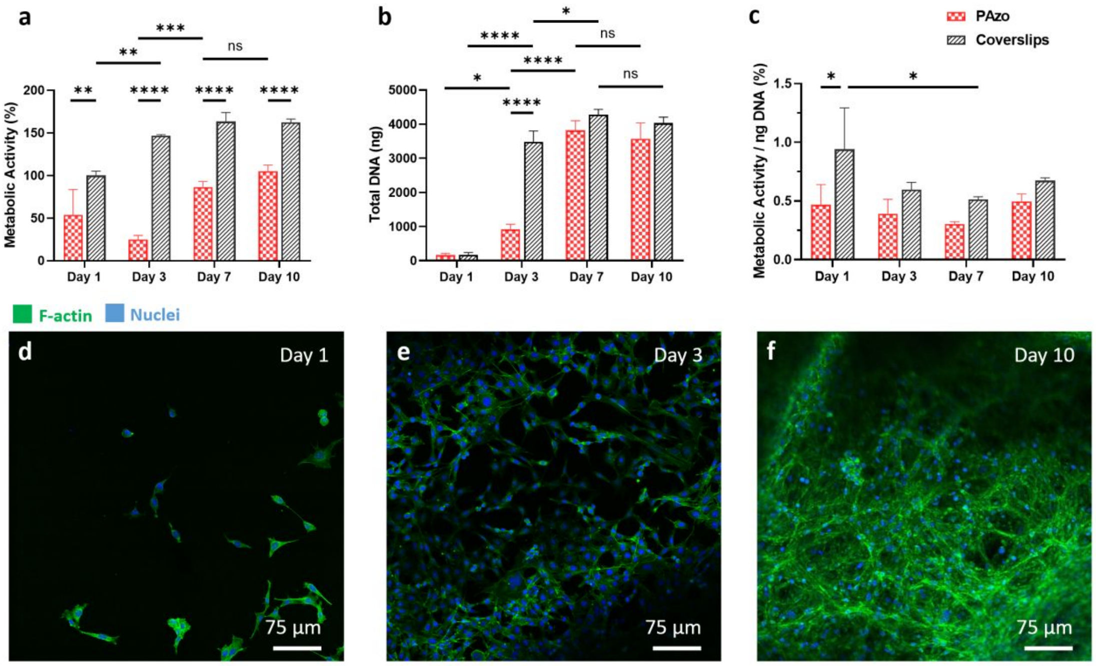

Figure S4. Cellular responses of NIH-3T3 fibroblast on PAzo film discs and glass coverslips as control: (a) Metabolic activities of 3T3 cells characterized by PrestoBlue assay; (b) Proliferation of 3T3 cells tested by total DNA assay; (c) Metabolic activities of 3T3 cells normalised by total DNA contents; (d-f) Immunocytochemical images of morphology (F-actin in green and nuclei in blue) of 3T3 cells on PAzo discs on Day 1, Day 3 and Day 10. (The difference between groups were analysed by two-way ANOVA with Turkey's post hoc test or two-tailed unpaired Student's t test. *p<0.05, **p<0.01, ***P<0.001, ****p<0.0001, ns = non-significance.)

To investigate the biocompatibility of PAzo, the embryonic mouse 3T3-J2 fibroblasts were seeded on the 2D PAzo discs and glass coverslips (as positive control), then evaluated cell viability and proliferation on day 1, 3 7, and 10. The cell morphology was observed to investigate the cell-substrate interaction via immunofluorescence confocal microscopy. The metabolic activities and proliferations of 3T3-J2 cells were quantified by PrestoBlue assay and Total DNA assay, respectively. In Figure S4a, the overall metabolic activity for the PAzo group dropped first due to the adaption of the new microenvironment in the initial days, and then increased significantly. As shown in Figure S4b, total DNA contents increased significantly in

the first 7 days in both PAzo and coverslips, followed by a plateau due to cell confluence. Figure S4c demonstrates the metabolic activity per ng DNA on PAzo discs and control groups, where cells on both substrates showed the highest values on Day 1, then witnessed a moderate decrease at early time points and rebounded at the later time point (Day 10). The initially high metabolic activity could be attributed to the adaptation of the new microenvironment right after seeding, while the rebound at later time point could be relevant to the stress of over confluence. In terms of the cell-substrate interaction, confocal microscopy confirmed cells attached and polarised during migration and then grew into a fully confluent morphology on the PAzo discs from Day 1 to Day 10 (Figure S4d-f), indicating good biocompatibility of PAzo promoting cell adhesion and proliferation.

UV-Vis absorption Spectra of PAzo in solution

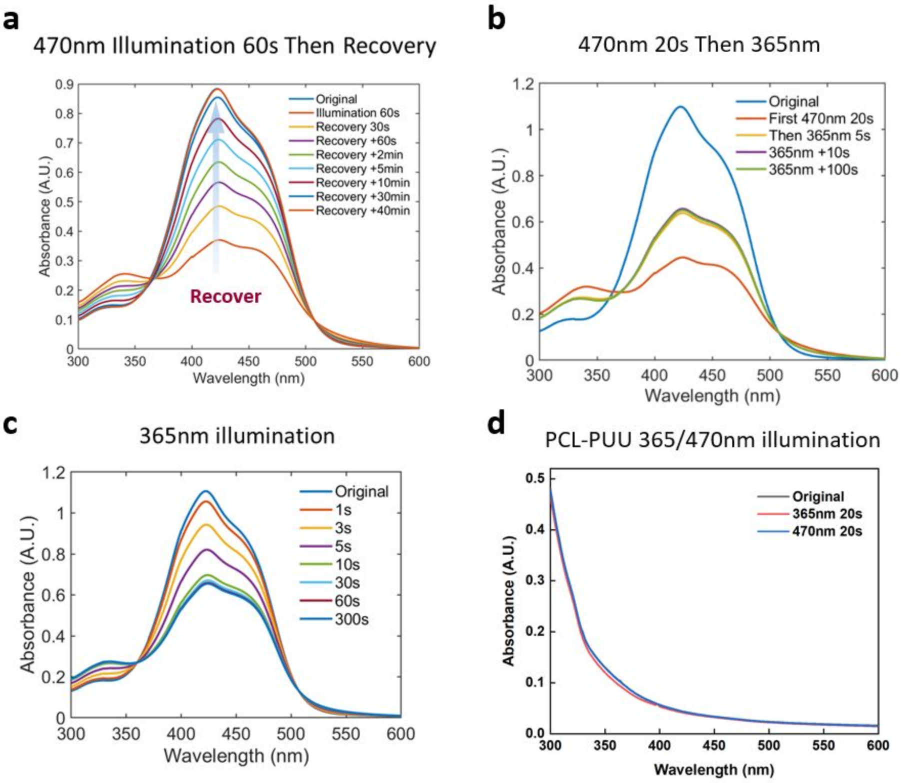

Figure S5. UV-Vis absorption spectra (deduct the background of DMAc solvent) (a) Recovery in the absorption of PAzo solution after the irradiation of 470 nm (low intensity) for 60 s; (b) Changes in the absorption of PAzo solution upon the irradiation with 470 nm light (0.73 W/cm^2) for 20 s then 365 nm UV light (0.73 W/cm^2) for different time; (c) Changes in the absorption of PAzo solution upon the irradiation with 365 nm light (0.73 W/cm^2) for different time period; (d) absorption of PCL-PUU (PCLU) under three conditions i.e., without light irradiation, 365 nm light irradiation for 20 s and 470 nm light irradiation for 20 s.

Wide/Small-Angle X-ray Scattering (WAXS/SAXS) of PAzo film.

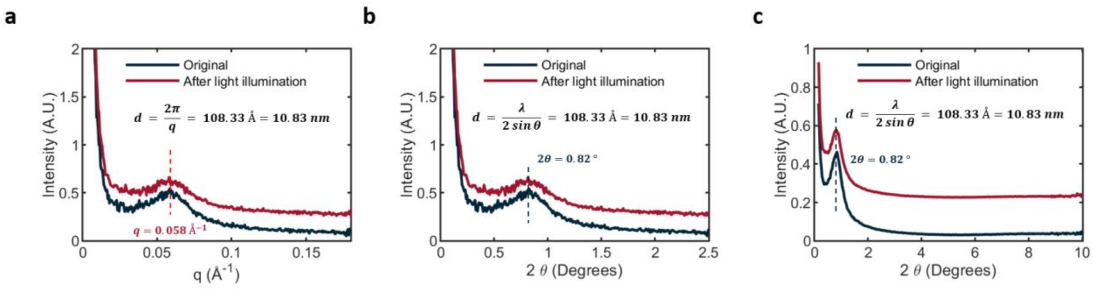

Figure S6. X-ray diffraction spectra of (a,b) Extra Small Angle X-ray Scattering (ESAXS) and (c) Middle Angle X-ray Scattering (MAXS) of PAzo films before and after light irradiation treatment.

Coefficient of Thermal Expansion (CTE) of PAzo determined by thermal contraction/expansion experiment

The thermal contraction/expansion test (Figure S7) was conducted using a DMA machine (DMA Q800, TA Instrument) following a reported method[3]. Briefly, a small stretching force of $1 \times 10^{-4}\,N$ was applied to ensure the sample was straight. The temperature was then decreased from 80 °C to 10 °C at a rate of 2 °C min^{-1} and heated back to 80 °C at the same rate. From Figure S7, the CTE of PAzo was determined to be $204.4 \times 10^{-6}\,℃^{-1}$ within the temperature range of approximately 12 °C to 32 °C, via linearly fitting the experimental data. This CET result was used for theoretical calculations and FEA simulations.

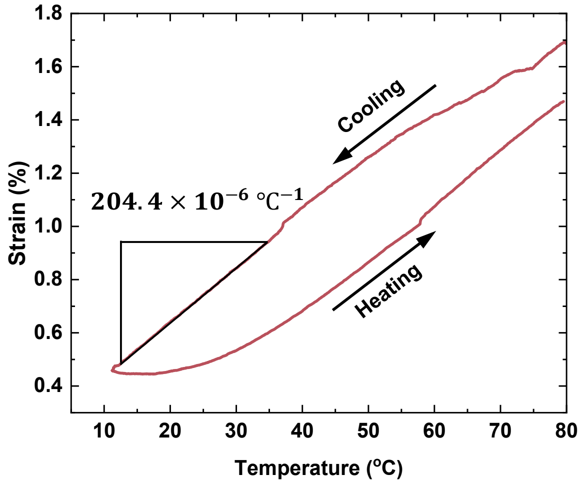

Figure S7. The experimental results of thermal contraction/expansion test for PAzo. (For determining CTE of PAzo)

Atomic Force Microscopy images of PCL-PUU

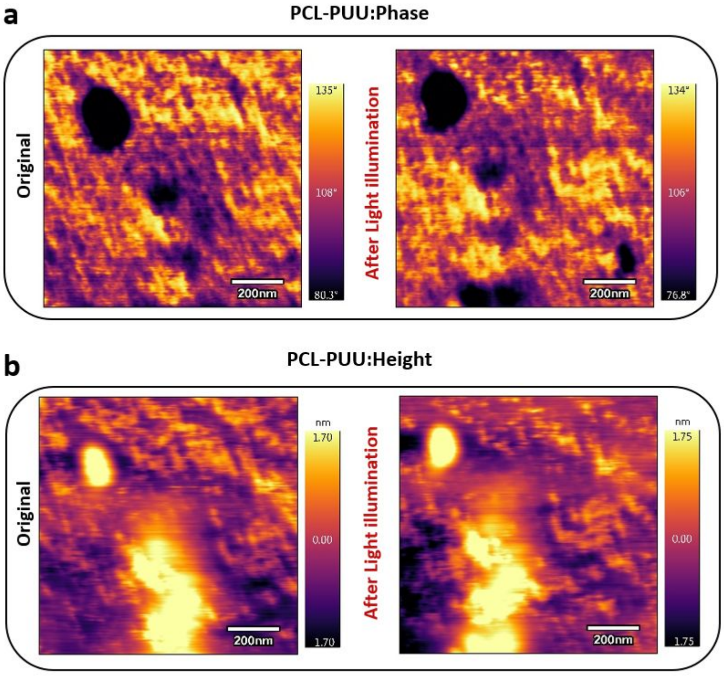

Figure S8. Ambient, tapping mode AFM images of the original PCL-PUU and PCL-PUU after light illumination treatment (a) Phase, and (b) Height.

The comparison of mechanical performances between PAzo and Kapton characterized via tensile tests

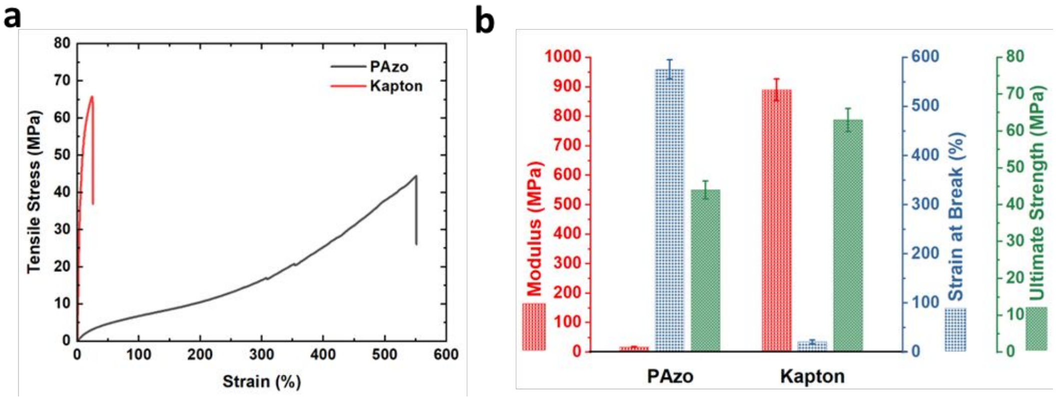

Figure S9. The comparisons of mechanical performance between PAzo and Kapton. (a) Stress-strain curves of PAzo and Kapton; (b) Tensile modulus, strain at break and ultimate strength of PAzo and Kapton.

The comparison of temperature changes among PAzo, PCLPUU and Kapton when irradiated by 470 nm LED light with different light intensities

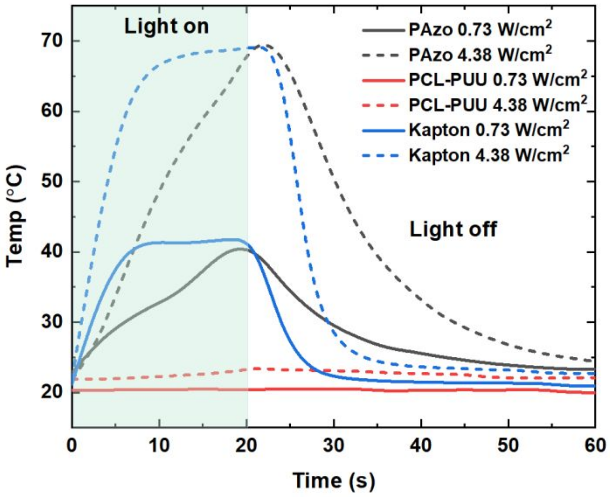

Figure S10. The comparison of temperature changes among PAzo, PCLPUU and Kapton when irradiated by 470 nm LED light with low and high intensity. The temperature of PCLPUU does not show an obvious change after irradiated upon 470 nm LED light irradiation with low and high intensities, While PAzo and Kapton show temperature changes of around 20 ℃ and 50 ℃ upon the irradiation of low intensity of 0.73 W/cm^2 and high intensity of 4.38 W/cm^2, respectively.

Bending angle and related temperature of PAzo/Kap bilayer actuator plotted with 100-cycle light stimuli, different light intensity and cycle period

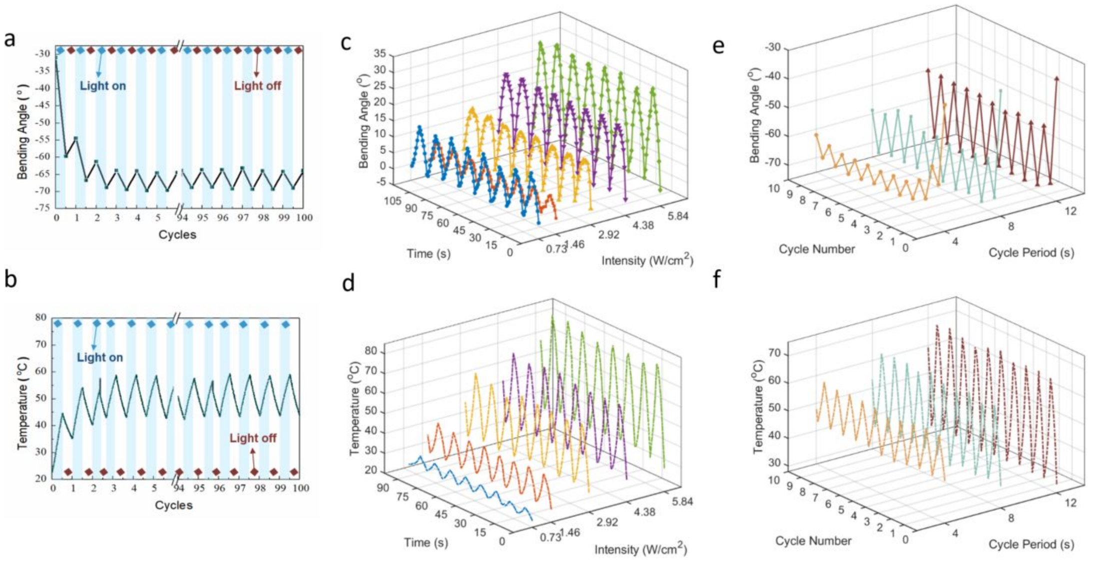

Figure S11. (a) Cyclic bending angle, (b) Cyclic temperature of bilayer actuator stimulated by periodic light stimulation (4.38 W/cm^2) for 2s on and 2s off in each cycle; (c) Bending angle (d) The highest temperature as a function of time by periodic light stimulation for 12s on and 12s off in each cycle with different intensities; (e) The highest bending angles (f) the highest temperature plotted as a function of light irradiation frequency (light cycle period) with a constant light intensity (4.38 W/cm^2) being periodically turned on and off.

The dynamic light-driven bending performance of the actuator were systematically characterised using the light intensity up to 4.38 W/cm^2 and cycle period of 4s (2s on, 2s off). To evaluate the repeatability of the light-driven actuator, we performed cyclic bending tests on the actuator, as shown in Figure S11a and b. These tests indicate that the actuator bending behaviour and related temperature changes are stable and repeatable during 100-cycle light stimuli, except for the 1st and 2nd cycles. The initial variation of the bending angle and temperature of the actuator in the 1st and 2nd cycles of warm-up may be due to the relaxation of internal stress induced by thermal history during fabrication of the actuator. Figure S11c and d display the cyclic bending angle and related temperature changes plotted with cycle time at different light intensity irradiation. As expected, the actuator deflected higher bending angles as the light intensity increased. With the same setup at a constant light intensity of 4.38 W/cm^2,

we tested the deflection and temperature of the actuator at different cycle period (i.e., frequency) as shown in Figure S11e and f. It is interesting to see that the time-dependence of photo-driven dynamic behaviour, i.e., the longer cycle of light stimulates, the larger bending angle is deflected, which is reminiscent of the characteristic time-temperature dependence of polymer viscoelasticity. Overall, these four figures show the fully recoverable actuation with excellent controllability and stability properties of the light-driven actuator.

Actuation stress of PAzo/Kap bilayer actuator as a function of light intensity, cycle period and thickness

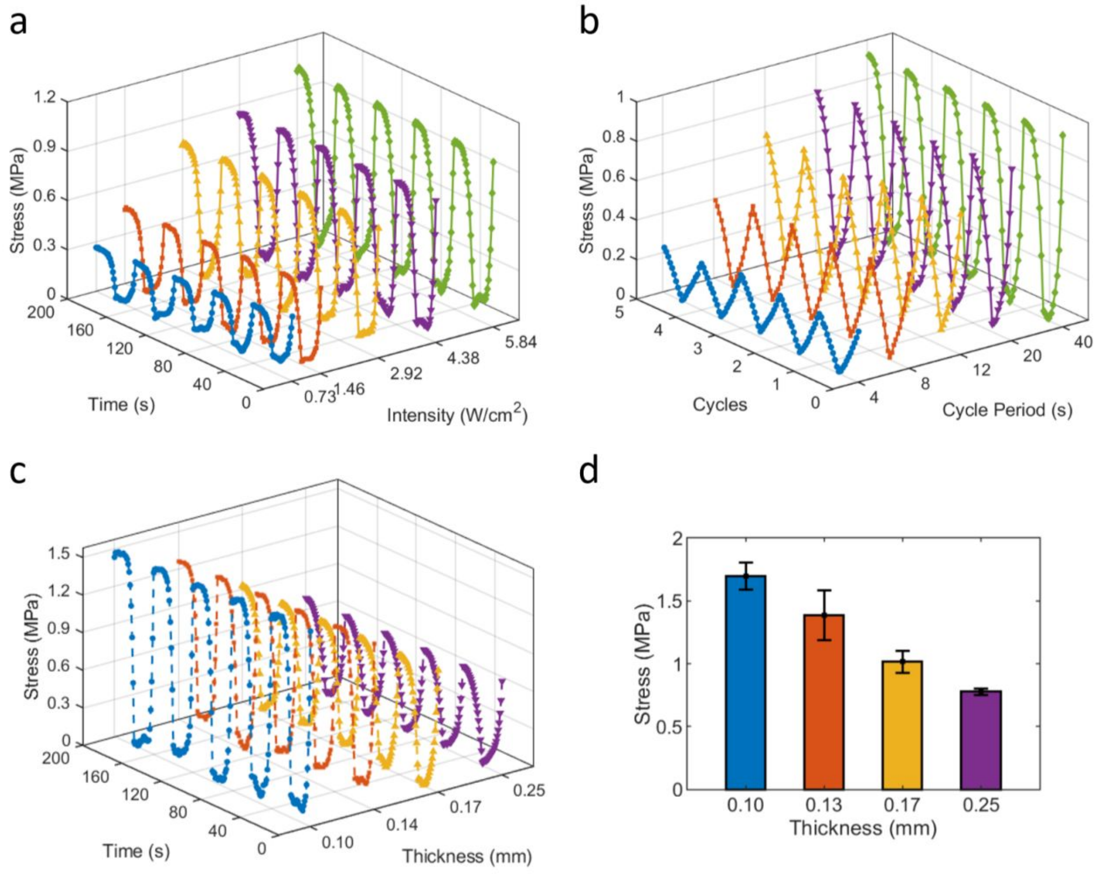

Figure S12. Actuation stress analysis of the PAzo/Kap bilayer (length of 10 mm, width of 2 mm, no specific statement, thickness: 0.16 mm, cycle period: 40s, light intensity: 4.38 W/cm^2) under the 470 nm light irradiation at different states. (a) Actuation stress of the bilayer as a function of cycle time and light intensity stimulated by periodic light for on (20s) and off (20s) (1% pre-strain); (b) Actuation stress as a function of irradiation cycle number and cycle period (frequency) stimulated by periodic light (4.38 W/cm^2); (c) Actuation stress as a function of cycle time and the thickness of PAzo/Kap bilayer actuator stimulated by period light (4.38 W/cm^2) for on (20 s) and off (20 s) (thickness of Kapton is kept at constant of 0.05 mm); (d) Actuation stress decreases with increasing the thickness of PAzo/Kap bilayer.

Force output stability of the bilayer actuator after 100 light on-off cycles

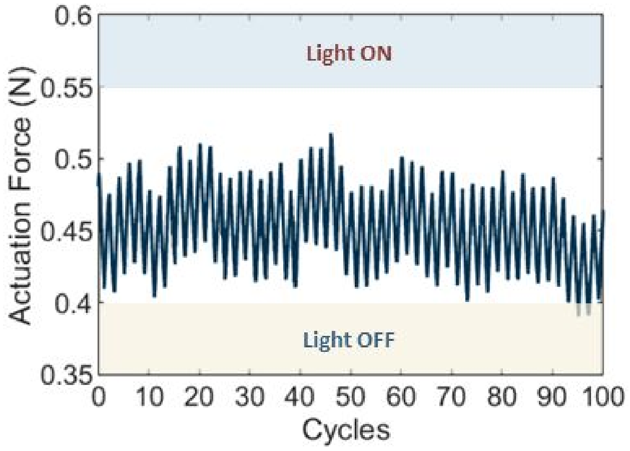

Figure S13. Force output of the PAzo/Kap under cyclic illumination of 470 nm LED light. (PAzo/Kap, light intensity of 4.38 W/cm^2, illumination period of 2 s ON and 2 s OFF, thickness of 0.25 mm).

Theoretical analysis of bending angle of bilayer actuator upon light irradiation with low intensity

Although we have explained the preliminary mechanisms of these two bending behaviours, theoretical analysis can help us better understand the bending behaviour under low-intensity light irradiation. Under low light irradiation with a low light intensity of 0.73 W/cm^2, the maximum temperature of the PAzo/Kap is around 30.4 ℃, which is lower than the melting temperature of 38 ℃ (Figure 2e). We make the first assumption that the photothermal effect of the bilayer dominates the bending behaviour under low light intensity irradiation.

Based on Timoshenko's theory of bending of a bimetal strip subjected to uniform heating[2], the general equation for the curvature of the PAzo/Kap bilayer upon light irradiation (Figure S14a) is

$$\frac{1}{\rho}=\frac{6(\alpha_2-\alpha_1)(t-t_0)(1+m)^2}{h(3(1+m)^2+(1+mn)(m^2+\frac{1}{mn}))} \tag{1}$$

$$m=\frac{a_1}{a_2} \tag{2}$$

$$n=\frac{E_1}{E_2} \tag{3}$$

$$h=a_1+a_2 \tag{4}$$

where ρ is the radius of curvature; α_1 and α_2 are the coefficients of expansion of the Kapton and PAzo, respectively; t_0 and t are the original and given temperatures, respectively; h is the thickness of the bilayer strip; m is the thickness ratio of Kapton layer (a_1) and PAzo layer (a_2); n is the Young's modulus ratio.

There are two more assumptions: 1) bending happens in the whole strip after light irradiation; 2) the strip is subjected to uniform heating when reaching the highest temperature.

$$\theta = \frac{360}{2\pi} \cdot \frac{L}{\rho} \tag{5}$$

where L is the arc length of the $\widehat{AC}$ (Here, L = 10 mm).

Combining equations (1)-(5), we obtained:

$$\theta = \frac{1080L(\alpha_2 - \alpha_1)m(1+m)}{\pi a_1(3(1+m)^2 + (1+mn)(m^2 + \frac{1}{mn}))} \Delta T \tag{6}$$

Hence, if α_2, α_1, m and n remain constant, the bending angle θ can only be regulated by ΔT (the change of temperature before and after light illumination). (Figure S15 shows the storage modulus of PAzo at various temperatures. As the temperature increases from 20 ℃ to 40 ℃, the storage modulus of PAzo exhibits a slight decrease, from 53 MPa to 37 MPa. Therefore, we assume that the modulus of PAzo remains relatively constant for theoretical calculations under low-intensity light illumination.) Substituting all parameters (in Table S4) into equation (6), when under light irradiation with low intensity ($\Delta T = 9$ ℃), $\theta \approx 6.14°$. The theoretical formula of the bending angle at different increased temperature is plotted in Figure S14b.

It is of note that the theoretically calculated angle is less than that by experiments (~ 20°), which indicates that photothermal mechanism is not the predominant contributor to the bending behaviour upon low intensity light irradiation. In other words, it is speculated that photochemical mechanism or the azobenzenes paly an essential role in the resulted bending angle.

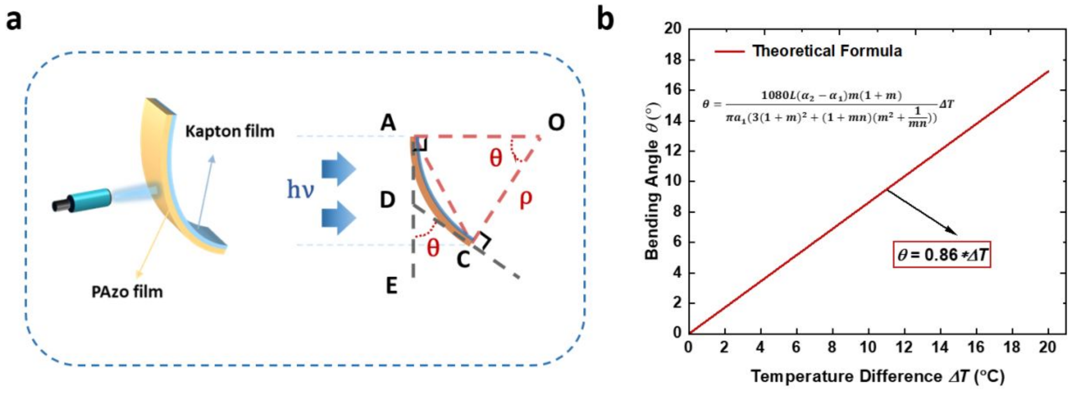

Figure S14. (a) Schematic diagram of double layer actuator in theoretical model; (b) Theoretical formula of bending angle plotted with the change of temperature for PAzo/Kap under 470nm light irradiation with low light intensity.

Energy conversion of PAzo/Kap bilayer actuators

The photothermal conversion efficiency (defined as the generated elastic energy accumulated during deformation divided by the incident light energy) of PAzo/Kap bilayer actuator was investigated, since energy loss must be considered when it turns into mechanical deflection. Follow the reported the calculation method[4] based on the bimetallic strip thermostat model.[2] The elastic energy stored in the actuator is defined by equation (7),

$$W_e = \frac{1}{2}\varepsilon_T \sigma_T \tag{7}$$

where ε_T and $\sigma_T$are the strain and stress during bending performance, which are depicted as follow (according to the reported model[5]):

$$\sigma_T = \frac{E_f E_s (\mathrm{H}_f + \mathrm{H}_s)[\mathrm{E}_s \mathrm{H}_s^2 (3\mathrm{H}_f + \mathrm{H}_s) + \mathrm{E}_f \mathrm{H}_f^2 (\mathrm{H}_f + 3\mathrm{H}_s)]}{E_f^2 + H_f^4 + E_s^2 H_s^4 + 2E_f E_s H_f H_s (2\mathrm{H}_f^2 + 2\mathrm{H}_s^4 + 3\mathrm{H}_f \mathrm{H}_s)} \varepsilon_T \tag{8}$$

$$\varepsilon_T = \frac{E_f^2 + H_f^4 + E_s^2 H_s^4 + 2E_f E_s H_f H_s (2\mathrm{H}_f^2 + 2\mathrm{H}_s^4 + 3\mathrm{H}_f \mathrm{H}_s)}{6E_f E_s H_f H_s (\mathrm{H}_f + \mathrm{H}_s)} k \tag{9}$$

$$W_e = \frac{[\mathrm{E}_s \mathrm{H}_s^2 (3\mathrm{H}_f + \mathrm{H}_s) + \mathrm{E}_f \mathrm{H}_f^2 (\mathrm{H}_f + 3H_s)][\mathrm{E}_f^2 \mathrm{H}_f^4 + \mathrm{E}_s^2 \mathrm{H}_s^4 + 2\mathrm{E}_f \mathrm{E}_s \mathrm{H}_f \mathrm{H}_s (2\mathrm{H}_f^2 + 2\mathrm{H}_s^4 + 3\mathrm{H}_f \mathrm{H}_s)]}{36 E_f E_s H_f^2 H_s^2 (\mathrm{H}_f + \mathrm{H}_s)} k^2 \tag{10}$$

The subscripts f and s represented the PAzo layer and Kapton layer, respectively. E refers to Young's modulus, H is the thickness and κ is bending curvature:

$$\kappa = \frac{1}{\rho} \tag{11}$$

The elastic energy of the bilayer actuator:

$$Q_{elastic} = W_e \cdot V_{volume} = W_e \cdot S_{sample} \cdot H \tag{12}$$

where S_{sample} is the surface area of the bilayer, H is the thickness of the bilayer.

Light input energy was calculated by:

$$Q_{lig\,ht} = \rho_{lig\,ht} \cdot S_{lig\,ht} \cdot t \tag{13}$$

where $\rho_{lig\,ht}$ is the light intensity and $S_{lig\,ht}$ is the light illumination area, t is the actuation time.

Finally, the energy conversion efficiency (η) is the ratio between elastic energy and light input energy:

$$\eta = \frac{Q_{elastic}}{Q_{lig\ ht}} \times 100\% \qquad (14)$$

We calculated the energy conversion efficiency of the PAzo/Kap actuator under low intensity (0.73 W/cm^2) 470 nm light irradiationto be around 0.02%, which is comparable to the reported efficiency of other light-driven bilayer actuators[4, 6].

Storage Modulus of PAzo at different temperature

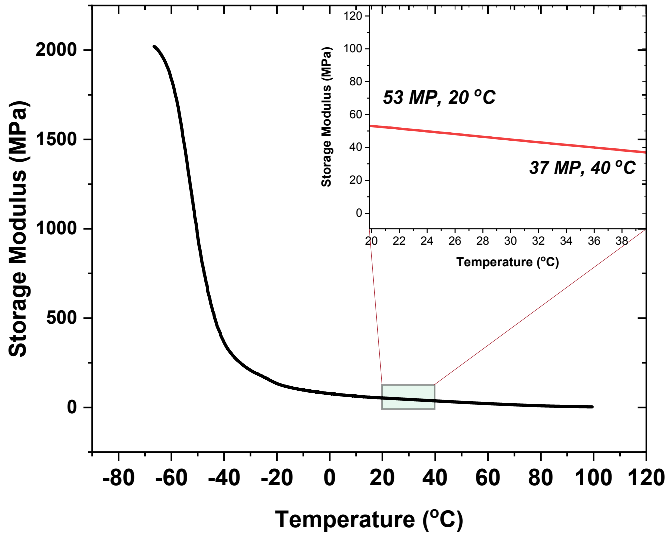

Figure S15. Storage Modulus of PAzo plotted with temperature.

Optical image of the control system for the application of soft robotic fingers to play piano

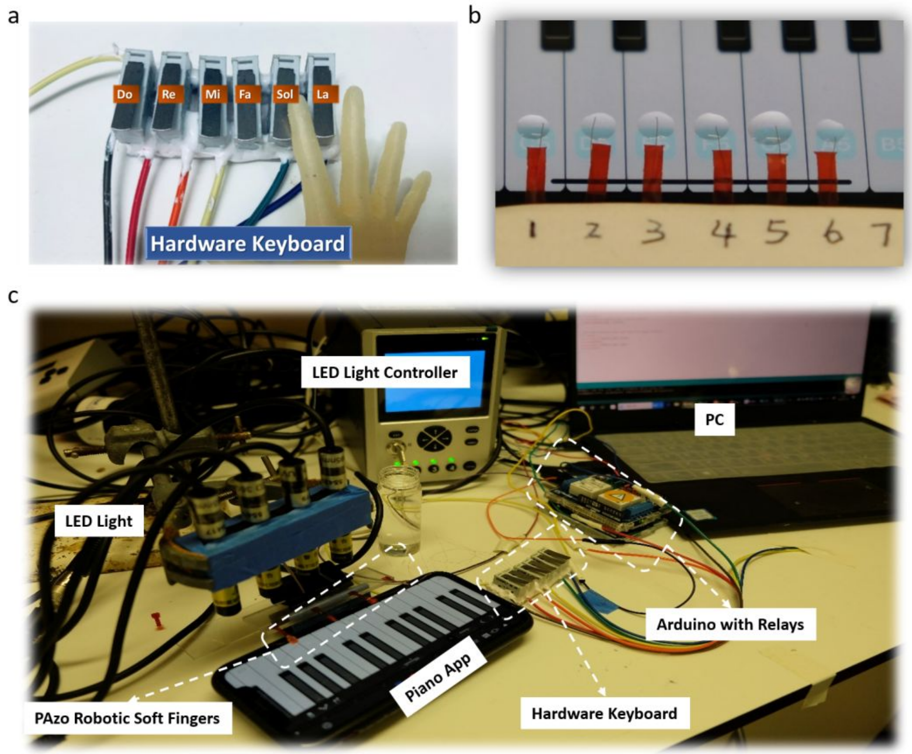

Figure S16. Optical images of (a) the home-made hardware keyboard, (b) robotic fingers connected to a bottle of saltwater via a thin and flexible copper wire, with a conductive salt droplet on each music note, and (c) the entire circuit and control system for the soft robotic fingers demo for playing the piano. The working principle of the robotic finger's ability to play music on iPhone is as follow: The PAzo/Kapton film actuator does not directly touch the piano keyboard. Instead, soft conductive copper wires are attached to the tips of the PAzo/Kap films, and salt drops are placed on each note of the keyboard. When the light is on, the actuator will bend, and its tip will touch the salt drop, removing ions and producing the note sound from the phone. Our "photo-finger" is not conductive and only serves the bending function to help the conductive wire touch the iPhone screen.

Table S1. Comparisons of light responsive materials reported in literatures.

**Material Type**	**Tg (℃)**	**Tm (℃)**	**Ti (℃)**	**Photo-mechanics Principle**	**Chain Architectures**	**Azobenzene Position**	**Modulus (MPa)**	**Strain at break (%)**	**Tensile Strength (MPa)**	**Actuation Stress (MPa)**	**Authors**
Graphene/PU	-36.5	41	-	Photothermal	Linear chain/Doping	-	10 - 22	~150	10 – 20	0.84	Liang et al. (2009) [7]
Graphene/PDMS	-	-	-	Photothermal	3D network/Doping	-	1.7 - 1.8	140 - 165	2 – 5	-	Leeladhar et al. (2017) [8]
CLCP	82/32	-	~140	Photochemical	3D network	Side-chain	42 – 48	~ 0.5	~1.3	~0.26 - 0.33	Liu et al. (2017) [9]
LCE-CNT composite	-	-	~100	Photothermal	3D network/Doping	-	5	~95	~11	-	Ahn et al. (2018) [10]
BA2DA-PVDF	-	-	-	Photochemical	Doping	-	~100 000	~0.015	~12.5	-	Yu et al. (2018) [11]
LCN/LCE	29 - 16	~ 54	60 - 125	Photochemical	3D network	Cross-linker	140 - 65.6	~30- 50	~17 – 25	-	Yue et al. (2018) [12]
GO/CNTs	-	-	-	Photothermal	-	-	160 – 20110	~2.5	~90 – 120	-	Li et al. (2019) [13]
Carbon black/PET	-	-	-	Photothermal	Doping	-	~2000 – 5000	~5	50 – 150	3.5	Li et al. (2019) [14]
Thermoplastic polyurethane	-25	-	-	Photochemical	Linear chain	Main chain	0.01 – 0.11	600 - 960	0.19 – 0.86	~0.010	Li et al. (2019) [15]
CPB-PE	-	25 - 70	-	Photothermal	3D network	-	19.65	~30	~3	-	Yang et al. (2019) [16]
LCN	-	-	-	Photochemical/Photothermal	3D network	Cross-linker	~400 – 1200	~1 – 3	~12 - 16	-	Zeng et al. (2019) [17]
PNIPAAM/AuNPs Hydrogel	-	-	-	Photothermal	3D network/Doping	-	0.0063 – 0.0273	12 - 63	~0.003 -0.006	-	Zhao et al. (2019) [18]
LCE	6.1 - 8.1	-	71.5-88.3	Photothermal/Photochemical	3D network	Doping	~0.1	~140	~0.09	-	Zuo et al. (2019) [19]
UHMW-PE/BZT	>RT	140	-	Photothermal	Linear chain/Doping	-	80000	~5	~100	70	Bhatti et al. (2020) [20]
PAANa/IONPs Hydrogel	-	-	-	Photothermal	3D network/Doping	-	~1	50	0.5	-	Li et al. (2020) [21]
AuNR/LCE	3 - 5	-	155-161	Photothermal	Linear chain	-	4.2 - 20.3	~40	1 - 3.7	-	Wang et al. (2020) [22]
CNT/PDMS	-	-	-	Photothermal	Doping	-	~50	120	~2	-	Yang et al. (2020) [23]

PU-Azo/Au	-14.89	60.10	111.6	Photochemical/Photothermal	Linear chain/Doping	Main chain/Doping	143.9 - 205.3	274.84	7.76 - 12.94	-	Chen et al. (2021) [24]
CNT-LCDANs	25	-	62 - 66	Photothermal	3D network/Doping	-	~200	~120	15-22.5	0.7 - 1.5	Jiang et al. (2021) [25]
LCN	-	-	50 - 78	Photothermal	3D network	-	1.5 - 40.9	~40	4	-	Xiao et al. (2021) [26]
Theromoplastic PSeDA	0	50 - 100	-	Photochemical/Photothermal	Linear Chain	Main chain	~20	~85	8	-	Xuan et al. (2021) [27]
LCE	1	-	95	Photothermal	3D network	-	~0.5	300 – 400	3 – 4	0.02 - 0.28	He et al. (2021) [28]
**Thermoplastic PAzo Elastomer**	**-54**	**38**	**-**	**Photochemical/Photothermal**	**Linear chain**	**Main chain**	**17.6**	**575.2**	**44**	**0.25 - 1.7**	**This study: Wu et al. (2023)**

Table S2. Thermal properties of PAzo before and after light irradiation

PAzo	T_g (℃)	$T_{c-onset}$ (℃)	T_{c-peak} (℃)	$T_{m-onset}$ (℃)	T_{m-peak} (℃)	ΔH_{cryst} (J/g)	ΔH_{melt} (J/g)
Original	−55.49	5.46	−7.95	32.94	38.62	3.08	18.82
After light illumination	−53.68	10.13	−1.94	32.11	38.82	12.81	23.64

Table S3. $\boldsymbol{d_{spacing}}$ of PAzo calculated from WAXD and SAXS results

2θ (Degree)	0.82	17.69	19.95
$d_{spacing}$ (nm) ($d_{spacing} = \frac{\lambda}{2sin\,\theta}$)	10.83	0.50	0.45

Table S4. Material properties of PAzo and Kapton for theoretical calculations

Property	PAzo	Kapton	Unit
Young's modulus	17.6e6	880e6	Pa
Poisson's ratio	0.39	0.34	1
Thickness	0.11	0.05	mm
Thermal Conductivity	0.15~0.30	0.12	$W \cdot m^{-1} \cdot K^{-1}$
Coefficient of Thermal Expansion (CTE)	204.4e-6	20e-6	K^{-1}

Reference

(1) Yildirimer, L.; Buanz, A.; Gaisford, S.; Malins, E. L.; Remzi Becer, C.; Moiemen, N.; Reynolds, G. M.; Seifalian, A. M. Controllable degradation kinetics of POSS nanoparticle-integrated poly(ε-caprolactone urea)urethane elastomers for tissue engineering applications. *Scientific Reports* **2015**, *1*, 15040.

(2) Timoshenko, S. Analysis of bi-metal thermostats. *Josa* **1925**, *11*, 233-255.

(3) Ge, Q. Thermomechanics of Shape Memory Polymers and Composites. *Doctor of Philosophy (PhD), Mechanical Engineering, University of Colorado at Boulder* **2012**.

(4) Han, B.; Zhang, Y. L.; Zhu, L.; Li, Y.; Ma, Z. C.; Liu, Y. Q.; Zhang, X. L.; Cao, X. W.; Chen, Q. D.; Qiu, C. W.; et al. Plasmonic-Assisted Graphene Oxide Artificial Muscles. *Adv Mater* **2019**, *31*, e1806386.

(5) Merced, E.; Tan, X.; Sepúlveda, N. Strain energy density of VO2-based microactuators. *Sensors and Actuators A: Physical* **2013**, *196*, 30-37.

(6) Dong, Y.; Wang, J.; Guo, X.; Yang, S.; Ozen, M. O.; Chen, P.; Liu, X.; Du, W.; Xiao, F.; Demirci, U.; et al. Multi-stimuli-responsive programmable biomimetic actuator. *Nat Commun* **2019**, *10*, 4087.

(7) Liang, J.; Xu, Y.; Huang, Y.; Zhang, L.; Wang, Y.; Ma, Y.; Li, F.; Guo, T.; Chen, Y. Infrared-Triggered Actuators from Graphene-Based Nanocomposites. *The Journal of Physical Chemistry C* **2009**, *113*, 9921-9927.

(8) Leeladhar; Raturi, P.; Kumar, A.; Singh, J. P. Graphene-polydimethylsiloxane/chromium bilayer-based flexible, reversible, and large bendable photomechanical actuators. *Smart Materials and Structures* **2017**, *26*, 095030.

(9) Liu, Y.; Wu, W.; Wei, J.; Yu, Y. Visible Light Responsive Liquid Crystal Polymers Containing Reactive Moieties with Good Processability. *ACS Appl Mater Interfaces* **2017**, *9*, 782-789.

(10) Ahn, C.; Li, K.; Cai, S. Light or Thermally Powered Autonomous Rolling of an Elastomer Rod. *ACS Appl Mater Interfaces* **2018**, *10*, 25689-25696.

(11) Yu, Q.; Yang, X.; Chen, Y.; Yu, K.; Gao, J.; Liu, Z.; Cheng, P.; Zhang, Z.; Aguila, B.; Ma, S. Fabrication of Light-Triggered Soft Artificial Muscles via a Mixed-Matrix Membrane Strategy. *Angew Chem Int Ed Engl* **2018**, *57*, 10192-10196.

(12) Yue, Y.; Norikane, Y.; Azumi, R.; Koyama, E. Light-induced mechanical response in crosslinked liquid-crystalline polymers with photoswitchable glass transition temperatures. *Nature Communications* **2018**, *9*, 3234.

(13) Li, H.; Wang, J. Ultrafast yet Controllable Dual-Responsive All-Carbon Actuators for Implementing Unusual Mechanical Movements. *ACS Appl Mater Interfaces* **2019**, *11*, 10218-10225.

(14) Li, J.; Zhang, R.; Mou, L.; Jung de Andrade, M.; Hu, X.; Yu, K.; Sun, J.; Jia, T.; Dou, Y.; Chen, H.; et al. Photothermal Bimorph Actuators with In-Built Cooler for Light Mills, Frequency Switches, and Soft Robots. *Advanced Functional Materials* **2019**, *29*, 1808995.

(15) Li, S.; Tu, Y.; Bai, H.; Hibi, Y.; Wiesner, L. W.; Pan, W.; Wang, K.; Giannelis, E. P.; Shepherd, R. F. Simple Synthesis of Elastomeric Photomechanical Switches That Self-Heal. *Macromol Rapid Commun* **2019**, *40*, e1800815.

(16) Yang, Q.; Peng, C.; Ren, J.; Zhao, W.; Zheng, W.; Zhang, C.; Hu, Y.; Zhang, X. A Near-Infrared Photoactuator Based on Shape Memory Semicrystalline Polymers toward Light

-Fueled Crane, Grasper, and Walker. *Adv Opt Mater* **2019**, *7*, 1900784.

(17) Zeng, H.; Lahikainen, M.; Liu, L.; Ahmed, Z.; Wani, O. M.; Wang, M.; Yang, H.; Priimagi, A. Light-fuelled freestyle self-oscillators. *Nat Commun* **2019**, *10*, 5057.

(18) Zhao, Y.; Xuan, C.; Qian, X.; Alsaid, Y.; Hua, M.; Jin, L.; He, X. Soft phototactic swimmer based on self-sustained hydrogel oscillator. *Science Robotics* **2019**, *4*, eaax7112.

(19) Zuo, B.; Wang, M.; Lin, B. P.; Yang, H. Visible and infrared three-wavelength modulated multi-directional actuators. *Nat Commun* **2019**, *10*, 4539.

(20) Bhatti, M. R. A.; Bilotti, E.; Zhang, H.; Varghese, S.; Verpaalen, R. C. P.; Schenning, A. P. H. J.; Bastiaansen, C. W. M.; Peijs, T. Ultra-High Actuation Stress Polymer Actuators as Light-Driven Artificial Muscles. *ACS Applied Materials & Interfaces* **2020**, *12*, 33210-33218.

(21) Li, M.; Wang, X.; Dong, B.; Sitti, M. In-air fast response and high speed jumping and rolling of a light-driven hydrogel actuator. *Nature Communications* **2020**, *11*, 3988.

(22) Wang, Y.; Dang, A.; Zhang, Z.; Yin, R.; Gao, Y.; Feng, L.; Yang, S. Repeatable and Reprogrammable Shape Morphing from Photoresponsive Gold Nanorod/Liquid Crystal Elastomers. *Advanced Materials* **2020**, *32*, 2004270.

(23) Yang, L.; Chang, L.; Hu, Y.; Huang, M.; Ji, Q.; Lu, P.; Liu, J.; Chen, W.; Wu, Y. An Autonomous Soft Actuator with Light - Driven Self - Sustained Wavelike Oscillation for Phototactic Self-Locomotion and Power Generation. *Advanced Functional Materials* **2020**, *30*, 1908842.

(24) Chen, C.; Liu, Y.; He, X.; Li, H.; Chen, Y.; Wei, Y.; Zhao, Y.; Ma, Y.; Chen, Z.; Zheng, X.; et al. Multiresponse Shape-Memory Nanocomposite with a Reversible Cycle for Powerful Artificial Muscles. *Chemistry of Materials* **2021**, *33*, 987-997.

(25) Jiang, Z.-C.; Xiao, Y.-Y.; Cheng, R.-D.; Hou, J.-B.; Zhao, Y. Dynamic Liquid Crystalline Networks for Twisted Fiber and Spring Actuators Capable of Fast Light-Driven Movement with Enhanced Environment Adaptability. *Chemistry of Materials* **2021**, *33*, 6541-6552.

(26) Xiao, Y.-Y.; Jiang, Z.-C.; Hou, J.-B.; Zhao, Y. Desynchronized liquid crystalline network actuators with deformation reversal capability. *Nature Communications* **2021**, *12*, 624.

(27) Xuan, H.; Guan, Q.; Zhang, L.; You, Z. Thermoplastic Photoheating Polymer Enables 3D-Printed Self-Healing Light-Propelled Smart Devices. *Advanced Functional Materials* **2021**, *31*, 2009568.

(28) He, Q.; Wang, Z.; Wang, Y.; Wang, Z.; Li, C.; Annapooranan, R.; Zeng, J.; Chen, R.; Cai, S. Electrospun liquid crystal elastomer microfiber actuator. *Science Robotics* **2021**, *6*, eabi9704.
